# Computational prediction of the molecular mechanism of statin group of drugs against SARS-CoV-2 pathogenesis

**DOI:** 10.1038/s41598-022-09845-y

**Published:** 2022-04-14

**Authors:** Dipanjan Ghosh, Debabrata Ghosh Dastidar, Kamalesh Roy, Arnab Ghosh, Debanjan Mukhopadhyay, Nilabja Sikdar, Nidhan K. Biswas, Gopal Chakrabarti, Amlan Das

**Affiliations:** 1 Department of Biotechnology, Dr. B. C. Guha Centre for Genetic Engineering and Biotechnology, University of Calcutta, 35 Ballygunge Circular Road, Kolkata, West Bengal 700019 India; 2 Guru Nanak Institute of Pharmaceutical Science and Technology, 157/F Nilgunj Road, Panihati, Kolkata, West Bengal 700114 India; 3 Department of Genetics, Institute of Genetic Engineering, 30, Thakurhat Road, Badu, Madhyamgram, West Bengal 700128 India; 4 National Institute of Biomedical Genomics, PO NSS, Kalyani, West Bengal 741251 India; 5 Human Genetics Unit, Kolmogorov Bhaban, Biological Sciences Division, Indian Statistical Institute, 203, BT road, Kolkata, West Bengal 700108 India

**Keywords:** Computational biology and bioinformatics, Drug discovery

## Abstract

Recently published clinical data from COVID-19 patients indicated that statin therapy is associated with a better clinical outcome and a significant reduction in the risk of mortality. In this study by computational analysis, we have aimed to predict the possible mechanism of the statin group of drugs by which they can inhibit SARS-CoV-2 pathogenesis. Blind docking of the critical structural and functional proteins of SARS-CoV-2 like RNA-dependent RNA polymerase, M-protease of 3-CL-Pro, Helicase, and the Spike proteins ( wild type and mutants from different VOCs) were performed using the Schrodinger docking tool. We observed that fluvastatin and pitavastatin showed fair, binding affinities to RNA polymerase and 3-CL-Pro, whereas fluvastatin showed the strongest binding affinity to the helicase. Fluvastatin also showed the highest affinity for the Spike_*Delta*_ and a fair docking score for other spike variants. Additionally, molecular dynamics simulation confirmed the formation of a stable drug-protein complex between Fluvastatin and target proteins. Thus our study shows that of all the statins, fluvastatin can bind to multiple target proteins of SARS-CoV-2, including the spike-mutant proteins. This property might contribute to the potent antiviral efficacy of this drug.

## Introduction

The COVID-19 pandemic caused by SARS-CoV-2 (Severe acute respiratory syndrome coronavirus 2, a novel coronavirus strain) has posed a severe threat to humanity. SARS-CoV-2, also referred to as 2019 novel coronavirus (2019-nCoV) or human coronavirus 2019 (hCoV-19)^1^, is a positive-sense single-stranded RNA virus responsible for the highly contagious severe acute respiratory syndrome (SARS) in humans^2^. By the first week of January 2022, over 304 million confirmed cases and over 5.4 million deaths had been reported globally^3^. The 30 Kb long single-stranded SARS-CoV-2 genome consists of seven genes oriented in the following order: ORF1a, ORF1b, S, OEF3, E, M, and N from 5’ to 3’ direction. The viral mRNA encodes 29 structural and nonstructural proteins (nsps), including ORF 1a/b polyprotein, spike (S) glycoprotein, envelope (E), membrane (M), and the nucleocapsid (N) protein^4^. The ORFs 1a and 1b code for the proteases 3Cl-Pro (also known as Mpro or the Main protease), and PL-Pro respectively, that further cleave the polypeptide into 16 nonstructural proteins. These structural proteins play pivotal roles in the entry and assembly of the virus particles in the host cells^6,7^. The replication/transcription machinery of the virus is mediated by two enzymes, namely the RNA-dependent R.N.A. polymerase (RdRp) or nsp12 and helicase or nsp13. While RdRP mediates the viral replication, the helicase catalyzes the unwinding of double-stranded RNA formed during replication and allows the next round of viral replication^8,9^. Most of these structural and nonstructural proteins of SARS-COV-2 share highly conserved functional domains with its predecessor strain SARS-CoV-2^5^ and may be targeted with the existing or novel antiviral agents^6^.

Genomic analysis of the available SARS-CoV-2 sequences across the globe revealed that the viral genome had acquired a specific mutation in S protein, which facilitated its entry and infectivity in host cells. Those variants were found to possess the D614G mutation, i.e., the replacement of the aspartate (D) with glycine (G) at the 614th amino acid of S protein, and became predominant across the globe, outcompeting the wild type strain^7^. Also, it was further revealed that the D614G mutation enhanced the infectivity of the virus in the host cells^8,9^. With the progression of pandemic, SARS-CoV-2 acquired additional mutations in S protein on the background of D614G mutation in different geographical regions and classified as B.1.1.7or *Alpha*, B.1.351or *Beta*, P.1 or *Gamma* and B.1.617 or *Delta* variant. Due to their higher transmissibility, mortality, and immune-escape properties, these variants are known as the variant of concerns (VOCs, Table 1).

**Table 1:** SARS-CoV-2 variants of concerns: defining mutations in the spike protein.

VOC	Country of Origin	Characteristic Spike mutations	Biological relevance
Alpha (B.1.1.7)	England	del69/70, del144/145, N501Y, A570D, D614G, P681H, T716I S982A, D1118H	Increased transmissibility, hospitalization risk, and minimal effect on vaccine efficacy
Beta (B.1.351)	South Africa	D80 A, D215G, 241/243del, K417N, E484K, N501Y, D614G, A701V	Increased transmissibility, hospitalization risk, mortality, immune escape property and might affect the vaccine efficacy
Gamma (P.1)	Brazil	L18F, T20N, P26S, D138Y, R190S, K417T, E484K, N501Y, D614G, H655Y, T1027I, V1176F	Increased transmissibility, hospitalization risk, and minimal effect on vaccine efficacy
Delta (B.1.617.2)	India	T19R, G142D, L452R, T478K, D614G, P681R, D950N, del157/158	Increased transmissibility, hospitalization risk, mortality, immune escape property and might affect the vaccine efficacy

The *Alpha* variant was first detected in England in September 2020, and by February 2021, it accounted for nearly 95% of SARS-CoV-2 transmission in England^10^. The Spike_*Alpha*_ protein consists of nine mutations, including two deletions and seven amino acid substitutions (four in S1 and three in S2) such as Δ69–70HV and Δ144Y deletions; N501Y, D614G, A570D, P681H, T716I, S982A, and D1118H^11^. The *Beta* variant was first detected in South Africa in October 2020, and by January 2021, it had spread to several other countries of Africa and Europe, Australia, and Asia^12^. Compared to the wild-type virus, the Beta variant consisted of nine defining mutations in the spike protein in addition to D614G, namely A701V, D215G, D80A, E484K, K417N, N501Y, ΔL242–244 deletions, R246I, and L18F^13^. The *Gamma* variant was first reported in March 2020 in Brazil^14^. The *Gamma* variant consists of multiple spike protein mutations, including K417T, E484K, and N501Y in the RBD region, L18F, T20N, P26S, D138Y, and R190S in the NTD region, D614G and H655Y at C terminus in S1 subunit, and V1176F and T1027I in S2 subunit^15^. The biologically important mutations in S_Gamma_ include the N501Y, E484K, and K417T. In late 2020, a new variant was detected in Maharashtra, India, and was named Delta variant or B.1.617. It consists of three sub-lineages: B.1.617.1, B.1.617.2, and B.1.617.3, of which the B.1.617.1 and B.1.617.2 were first detected in India in December 2020, whereas the B.1.617.3 sub-lineage was first reported in India in February 2021^16^. Further studies revealed that B.1.617.2 is responsible for higher risk of transmissibility and hospitalization than the other sub-lineages^17^. The Spike Delta(B.1.617.2) consists of characteristic mutations such as T19R, G142D, L452R, T478K, D614G, P681R, D950N, and del157/158 (Outbreak.org, accessed on 20th October 2021).

The first three variants possess the mutation in RBD of S protein, characterized by the replacement of asparagine (N) with tyrosine (Y) at position 501 of RBD, on the background of D614G mutation^18,19^. The N501Y mutation has been reported to enhance the binding of viral S protein to the host ACE2 receptors and reduce the efficacy of the neutralizing antibodies targeting the RBD^20,21^. During the interaction of the neutralizing antibodies with RBD, the E484 residue plays an important role by forming hydrogen bonds and salt‐bridge interactions. The E484K mutation inhibits the formation of H‐bond and salt‐bridge interaction between the antibody and RBD and results in the reduced effectiveness of the neutralizing antibodies^22^. The *Beta* and *Gamma* variants shared three common mutations in their RBD, K417N/T, E484K, and N501Y, which may change their antigenic profile and reduce the efficacy of the neutralizing antibodies. Similarly, the P681H mutation (furin cleavage site) in the *Alpha* variant is located proximal to the spike antigenic sites and may adversely affect the neutralization efficiency of the antibodies^22^. The Delta -B.1.617.2 lineage possesses the signature mutations L452R, D614G, and P681R in the spike protein. The L452R mutation has been associated with reduced neutralization efficacy by antibody or vaccine, while P681R has been associated with enhanced transmissibility^23,24^. Thus these accumulating variations had enhanced the immune escape potential of new SARS-CoV-2 variants.

Since the prevailing vaccines were designed against the wild-type SARS-CoV-2 discovered in 2019, concerns have been raised about whether these vaccines will be effective against the new VOCs.The growing concern is that some of these emerging mutations reside in the antigenic supersite in the NTD and the RBM on RBD, which are the potential targets for neutralizing antibodies. A recent study indicated that B.1.1.7 is resistant to neutralization by most of the monoclonal antibodies targeting the N-terminal domain (NTD) of S-epitopes and also partially resistant to a few RBD-targeting antibodies. However, convalescent plasma or vaccine sera had no significant effect on the neutralizing efficacy against the *Alpha* variant. The B.1.351or *Beta* variant also showed increased resistance to neutralization by most of the monoclonal antibodies targeting NTD and RBM on RBD of spike proteins^25^. The vaccine trials conducted in South Africa for the AstraZeneca, Novavax, and Johnson & Johnson’s Janssen vaccines revealed lower vaccine efficacy in places where B.1.351 is dominant^26^. Other studies also reported a lower vaccine efficacy rate for Moderna^27^, Pfizer^28^, and BNT162b2^29^ against the *Beta* variant. Neutralizing efficiency of the vaccines against the Delta variant indicated a mixed response so far. It was reported that two doses of the BNT162b2 vaccine had given protection from the Delta (B.1.617.2) infection^30^. A recent study also revealed that the BNT162b2 vaccine was more effective than the ChAdOx1nCoV-19 against the *Delta* variant^31^. Thus the available reports suggest that the commercially available vaccines had a mixed spectrum of efficacy against the VOCs.

Hence the search for novel drug candidates which can target both the wild type as well as mutant proteins warrants investigation on utmost priority. Presently there are no well-defined effective therapies against COVID-19. Till December 2021, the only medicine prescribed by the doctors is dexamethasone, which has been shown to reduce 28-day mortality in COVID-19^32^. Although Remdesivir, a novel nucleotide analog, initially showed some promising results in lowering the oxygen requirement of hospitalized COVID-19patients^33^, later WHO has recommended against the use of remdesivir^34^. Very recently, FDA authorized Pfizer’s Paxlovid (nirmatrelvir and ritonavir tablets, co-packaged for oral use) for the treatment of mild-to-moderate COVID-19 patients^35^. Also, a plethora of randomized trials are going on to investigate the possible therapeutic remedies against COVID-19, but no conclusive outcome has emerged to date. By molecular docking and dynamics simulation approaches, several drug candidates were proposed that can target the essential proteins of SARS-CoV-2 like helicase, M-protease, and RdRP, to name a few. Compounds like cepharanthine, lumacaftor, cordycepin, pritelivir, etc., were found to target the helicase protein^36,37^. Similarly, several compounds showed good affinity towards the M-protease, including the natural compounds such as salvianolic acid A, curcumin^38^, the antivirals such as TMC-310911, and ritonavir^39^, terpenes^40^, and Rutin and flavone analogs^41^, to mention a few.

The beneficial effects of the statin group of drugs are well documented. The statins have shown a promising therapeutic role against various autoimmune inflammatory induced disorders such as multiple sclerosis, systemic lupus erythematosus, rheumatoid arthritis, etc.^42^. They also possess anti-hyperlipidemic and cardioprotective properties^43,44^. Moreover, statins have also been effective against several viral infections such as Avian influenza, Zika virus, hepatitis C virus, H1N1 pandemic, and the Ebola outbreak in West Africa^45–49^. In an interesting case study published in August 2020, a positive association between statin usage and reduced mortality of COVID-19 patients was first reported^50^. It was observed that the antecedent use of statin in hospitalized COVID-19 patients is associated with the lowering of mortality. At a similar timeframe, another retrospective case–control study with the COVID-19 patients hospitalized at Hubei province, China, reported that the overall mortality risk had significantly reduced in the patients with in-hospital use of statins, mainly atorvastatin^51^. In a retrospective single-center study, Daniels et al. published that statin use before hospitalization was associated with a substantially lower risk of COVID-severity and was also associated with a faster recovery^52^. Another multi-centric retrospective cohort study on the COVID-19 positive old population living in Belgiumrevealed a statistically significant association between statin usage and reduced COVID-19-severity^53^. In a recent report, Gerold et al. demonstrated the direct effect of statins on coronavirus infection in ex vivo conditions using the human lung cells^54^. They observed that among all the statins, fluvastatin significantly reduced the entry of SARS-CoV-2 into the human respiratory cells by modulating the host gene expressions^54^. Interestingly, they also observed that fluvastatin treatment significantly decreased SARS-CoV-2 genome copy numbers. But none of those mentioned above studies had demonstrated the direct effect of statins on SARS-CoV-2 target proteins. Hence the mechanism by which statins may inhibit viral pathogenesis remains inconclusive. In the present study by in silico molecular docking and molecular dynamics simulation analysis of the interactions of the statin group of drugs with the essential structural and functional proteins of SARS-CoV-2, we have aimed to predict a possible mechanism by which the statins may inhibit SARS-CoV-2 infection.

## Materials and methods

### Selection of target proteins of SARS-CoV-2 and its sequence homology with other coronaviruses

As discussed earlier, the spike (S)-protein, RNA dependent RNA polymerase (RdRp), 3-Chymotrypsin-like protease (3CL-Pro)or the main protease and helicase are the critical proteins that regulate the various stages of SARS-CoV-2 infection, including the entry and replication of the viral genome in the host cells^5,8,50,51,55,56^. Hence for this study, the crystalline structures of RdRp (PDB ID: 7BV2), 3CL-Pro (PDB ID: 6LU7), and helicase (PDB ID: 6ZSL)were obtained from the RCSC Protein Data Bank (https://www.rcsb.org).

The FASTA sequences of the target proteins related to SARS-CoV and coronaviruses from other species were derived from the UniProt database (https://www.uniprot.org/) for the sequence homology analysis. Multiple sequence alignment studies were performed with Clustal Omega (https://www.ebi.ac.uk/Tools/msa/clustalo/) to get the sequence homology data and generate the phylogenetic tree.

### Determination of the mutational landscape of SARS-CoV-2 genome

A total of 5,053,231 SARS-CoV-2 genome sequences deposited to GISAID until November 2021 were obtained and processed for downstream analysis. Sequences with an inadequate length (< 25,000 nt) and duplicated entries were excluded from the dataset. Sequences were mapped against the SARS-CoV-2 reference sequence (Wuhan/WH01/2019) using the NextAlign aligner tool (https://github.com/nextstrain/nextclade). To predict the time-dependent emergence of a specific mutation, we performed a phylodynamic analysis of global subsamples of SARS-CoV-2 sequences (N = 3525) using the next train/ncov pipeline (https://github.com/nextstrain/ncov). Entropy for each position on SARS-CoV-2 Spike protein, helicase, RdRp, and main protease was calculated using a method encoded in the nextstrain/ncov pipeline (https://github.com/nextstrain/ncov).

### Homology modeling, energy minimization, and validation of wild type and spike mutants

The sequence of SARS-CoV-2 spike protein was downloaded from the NCBI (https://www.ncbi.nlm.nih.gov/) protein database (Accession No: YP_009724390). Mutations were collected from the website, https://outbreak.info/situation-reports, accessed in October 2021. The template sequence was identified via alignment of the available PDB sequences using the BLASTp program (NCBI). All the sequences, including the wild type and mutants, were modeled using the Swiss Model Server^57^. Predicted structures were subjected to analysis in SWISS-MODEL for QMEAN Z-score calculation which includes cumulative Z-score of Cβ, All atoms, Solvation, and Torsion values. The templates for each mutated sequence were identified through searching on PDB using the BLASTp program provided by NCBI blast for all proteins. Due to the unavailability of the crystal structure of the region of interest, we have taken EM structure as a template for each different mutation based on query coverage, highest GMQE score, and percent (%) identity. The predictions were finally validated by PROCHECK. The summation of the number of residues in favored regions and allowed areas was considered for percent (%) quality assessment using the Ramachandran plot in PROCHECK (Table 2). For each of the models, a short molecular dynamics simulation of 25 ns of equilibrium was performed using Desmond simulation package v6.2 of Schrödinger LLC.

**Table 2:**
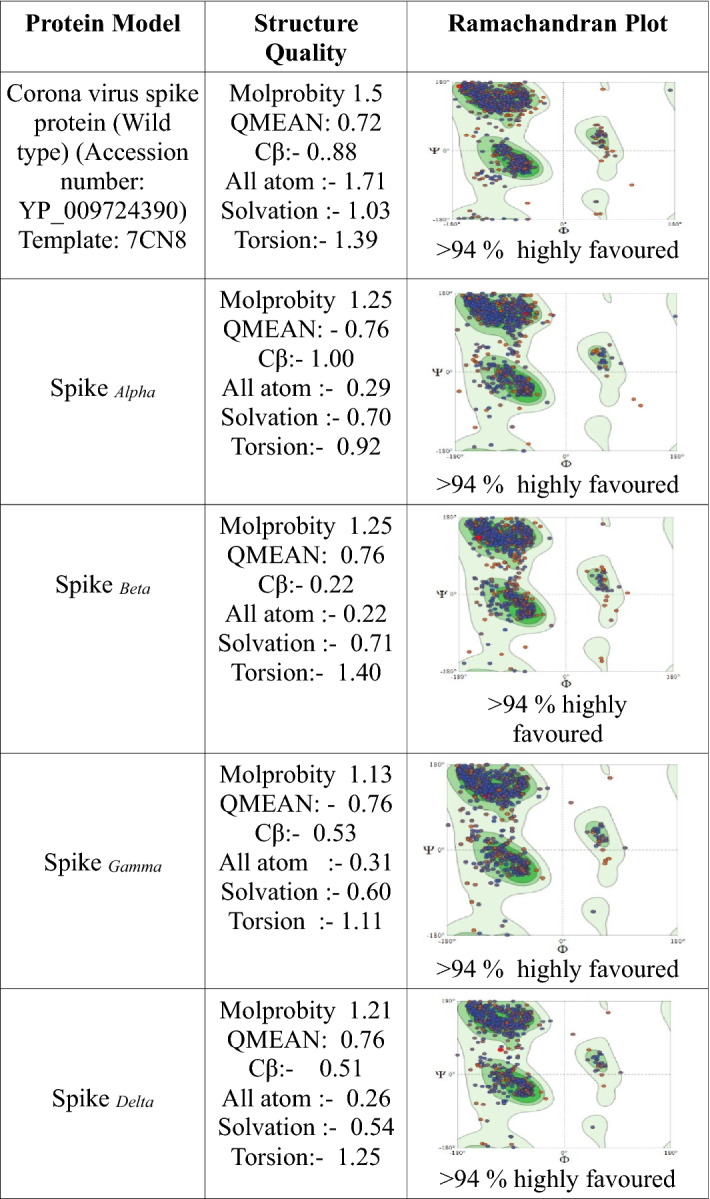
Modelling, energy minimization, and structure validation of wild type and mutant spike proteins.

### Determination of topology and structural alterations of wild type and mutant spike proteins

After modeling the wild-type and mutant spike protein, we evaluated the effects of the point mutations on different structural parameters, such as topology, folding dynamics, and stability. To determine the protein topology of the spike protein, we have used PredictProtein web-server (https://predictprotein.org/) and use the default setting therein. For further details about the secondary structure, we also used the SOPMA prediction web-server (https://npsa-prabi.ibcp.fr/cgi-bin/npsa_automat.pl?page=/NPSA/npsa_sopma.html). For isoelectric pI, aliphatic index, and protein instability index value determination, we have used https://web.expasy.org/protparam/.

Protein motifs were scanned using the webserver https://prosite.expasy.org/scanprosite/. Protein folding rate was measured using the FOLD-RATE web-server (https://www.iitm.ac.in/bioinfo/fold-rate/)^58^.

The tertiary structure of the protein was determined using the RoseTTAFold^59^. RoseTTAFold uses a machine learning-based method and includes a three-track network to process sequence, the distance between atoms, and coordinate information to predict the protein structure. For our study, we used the FASTA files of amino acid sequences of spike proteins (WT and variants) as input and then assigned in the server. The confidence score lies between 0–1, which corresponds to a local superposition-free score to compare protein structures. Structures with the highest score (> 0.6) were considered for analysis.

### Screening of ligand molecules using Schrödinger suite

#### Preparation of the ligand molecules

**Figure 1:**
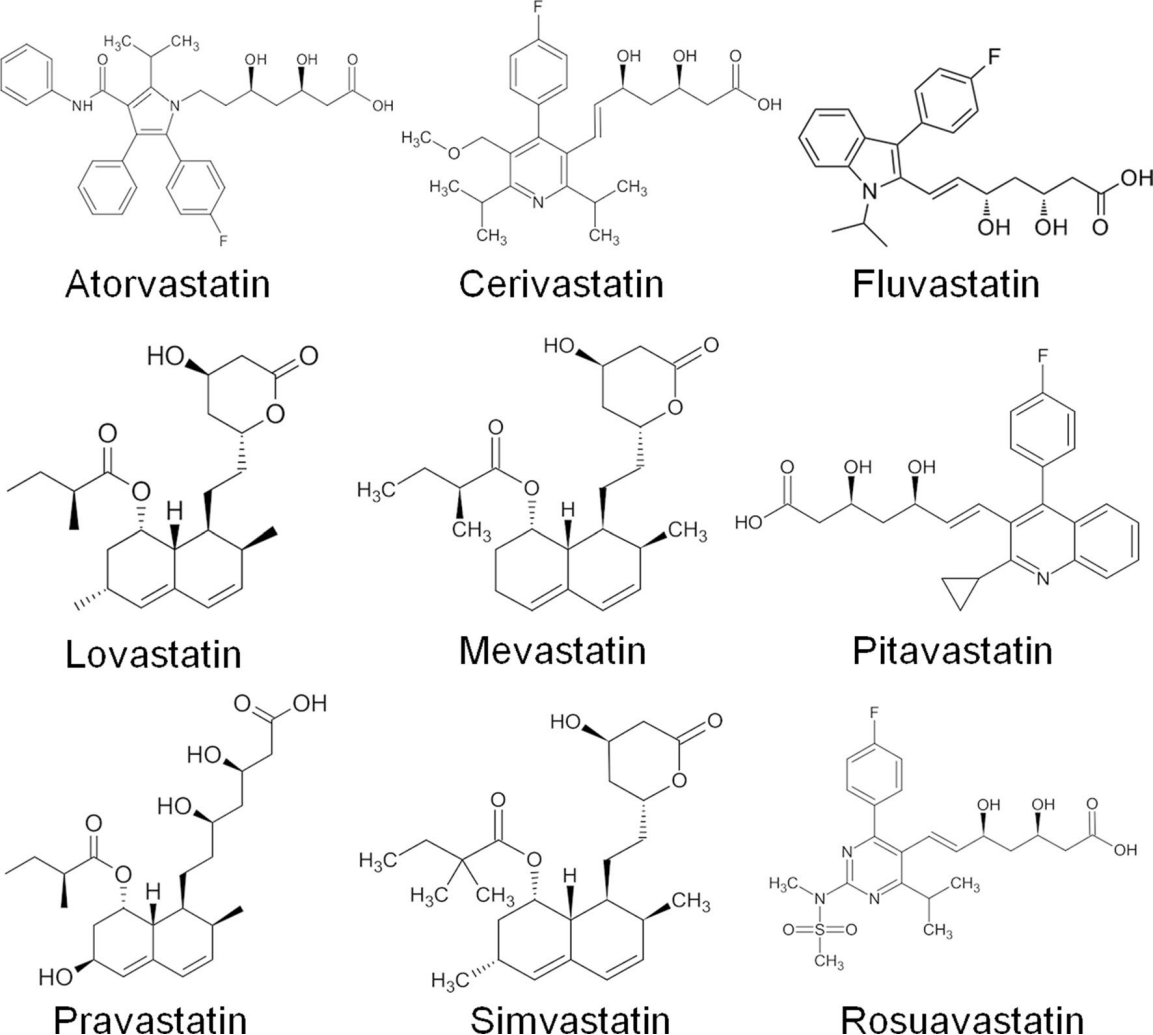
2D structure of the statin molecules.

Three-dimensional structures of nine statin groups of drugs (Atorvastatin, Cerivastatin, Fluvastatin, Lovastatin, Mevastatin, Pitavastatin, Pravastatin, Rosuvastatin, and Simvastatin) were obtained from PubChem database (https://pubchem.ncbi.nlm.nih.gov) (Fig. 1). The physicochemical properties of stains are listed in Table S1.To make the statins suitable for docking, we performed the following steps such as salt removal, the addition of H-atoms, and deprotonation using the LigPrep v4.7 module of the Schrodinger suite. Epik v4.7 module was used for charge neutralization of the drug candidates for attaining the biological relevant pH (pH 7.0 ± 0.2). The high-energy tautomeric states were excluded to retain up to four stereoisomers, and generation of only one stereoisomer per ligand was allowed. The best drug candidate(s) for different target proteins was predicted by the virtual screening workflow module of Maestro^60^.

#### Preparation of the target proteins

The target proteins obtained from the PDB database or modeled in other platforms required further processing for being used for the docking analysis. The non-standard residues(residues other than amino acids) were first removed from the target proteins using DS client, and the resulting protein structures were processed using the ‘protein preparation wizard’ of Maestro, Schrodinger^61^. The target proteins were processed by the addition of H-atoms, assigning the bond order using the CCD (Cambridge Crystallographic Datacenter) database, and introducing the missing disulfide linkages. The structures were further optimized for H-bond assignment at pH 7.0 using the PROPKA function. It was followed by a restrained energy minimization module by applying the OPLS3e force field to converge heavy atoms to an RMSD value of 0.30 Å^61^.

#### Grid generation

The grid generation is a very crucial step for the analysis of drug-target protein interaction. In this process, a 3-D boundary for the ligand-binding site was generated in the target protein using Glide, version 8.2 of Maestro, Schrodinger^62^. The top-ranked potential binding sites were identified by keeping at least 15 points per site for the generation of the standard grid. The ‘receptor grid generation module’ was used to generate grids of 20 Å size across the binding site.

#### Molecular docking

The molecular docking for screening of ligands (statins) was performed with the Glide Virtual Screening Workflow (VSW). It consisted of flexible docking in three successive modes, namely, the high throughput virtual screening (Glide HTVS), the standard precision (Glide SP), and the extra precision (Glide XP)^63^. The scaling factor was set to 0.80, and the partial charge cutoff was 0.15. At the end of Glide HTVS, 50% of compounds were retained with all possible states. They were subjected to docking with Glide SP mode with retention of 50% of the best compounds with a good scoring state. Finally, Glide XP docking was performed with these compounds with 100% retention to find the best scoring state. The parameters such as Glide score, Glide energy, Glide emodel were noted. Post docking binding-site analysis and generation of graphical representations were done with Maestro 12.5.139, Discovery Studio Client, version 16.10 (Accelrys Software Inc. San Diego)^64^ and VMD. (Visual Molecular Dynamics)^65,66^ software.

### Determination of MM-GBSA score

The binding energies of the statins with the target proteins were estimated as the molecular mechanical-generalized Born surface area (MM-GBSA) score, using the Prime MM-GBSA module of Schrodinger. The VSGB solvation model (Variable-dielectric generalized Born model)^67^ and OPLS3e force field were used in the process. The protein was kept rigid, while the ligand was considered flexible. The binding energy was calculated using the following equation:

1 $${\text{MM}} - {\text{GBSA }}\left( {\Delta {\text{G binding}}} \right) \, = {\text{ Energy of Optimized Complex }} - {\text{ Energy of Optimized Receptor }} - {\text{ Energy of Optimized Ligand}}$$

### Molecular dynamics simulations

Molecular dynamics (MD) simulations provided an insight into dynamic perturbations within the complex and interactions of ligand, lipid, and water molecules. All molecular dynamics simulations were done with the academic version of Desmond simulation package v6.2 of Schrödinger LLC^68^. Dual Intel Xeon Gold 6248-40 Core-80 thread processor with 384 GB RAM and Dual NVidia Tesla V100 GPU was used to run Desmond. The Protein–ligand complex was first solvated in an orthorhombic box using the Simple Point Charge model (SPC water model) of water that extends up to 10 Å from the protein^69^. The system was neutralized with 0.15 M NaCl. Periodic boundary conditions were applied to the system in the NPT (constant particle number, pressure, and temperature) ensemble using a Nose–Hoover chain thermostat with a relaxation time of 1.0 ps^70^. Martyna − Tuckerman − Klein with Isotropic coupling style was used as the barostat system with a relaxation time of 2.0 ps^71^. The temperature was set to 300 K, the pressure was maintained at 1.01325 bar, and the pH was maintained at 7.00 throughout the simulation process. The OPLS_20059 force field parameters were used^72^. The particle mesh Ewald method was used to calculate the long-range electrostatic interactions^73^. The r-RESPA (reversible reference system propagator algorithms) integrator with an integration time of 2 fs was used for the analysis^74^.

The system was initially set for a 100 ps run (5000 steps) using a hybrid method of the steepest descent and LBFGS (Limited memory Broyden-Fletcher-Goldfarb-Shanno) algorithm. It was followed by 25-ns equilibration with NVT (constant particle number, volume, and temperature) setting. During equilibration, the ligand was restrained while the protein was allowed to relax. Restraints were removed for subsequent production runs at 300 K and 1.01325 bar with an integration time step of 2 fs. Five hundred frames were collected at intervals of 400 ps throughout the 200 ns production run. The ligand–protein interactions were analyzed using the Simulation Interaction Diagram tool implemented in the Desmond MD package. The Protein RMSD, ligand RMSD, and the Root Mean Square Fluctuation (RMSF) were monitored throughout the simulation period to study the changes in structural conformation of the protein–ligand complex. RMSD and RMSF were calculated using the following equations:

2 $$RMSD_{X} = \sqrt{\frac{1}{N} \sum\limits_{i = 1}^{N} {(r^{\prime}_{i} (t_{X} )} ) - r_{i} (t_{ref} ))^{2}}$$

3 $$RMSF_{i} = \sqrt{\frac{1}{T} \sum\limits_{t = 1}^{T} { \langle (r^{\prime}_{i} (t))} - r_{i} ((t_{ref} ))^{2} \rangle }$$

where t_ref_ is the reference time, r_i_ is the position of residue i, r'_i_ is the position of atoms in the residue i, after superposition on the reference where frame x is recorded at time t_x_, and the angle brackets indicate that the average of the square distance is taken over the selection of atoms in the residue.

Solvent Accessible Surface Area (SASA) and Polar Surface Area (PSA) of ligand molecule was also monitored throughout the simulation period.

## Results

### Evolutionary relationship of therapeutically targeted SARS-CoV-2 proteins with other viral strains

The evolutionary conservancy is a critical feature of a gene or protein which guides it to act as a potential drug target. Hence the determination of interspecies evolutionary distance is necessary to predict the effectiveness of the candidate protein to become a potential drug target. In this study, we investigated the interspecies divergence of four selected target proteins of SARS-CoV-2, namely the spike protein, RdRp, helicase, and main protease among the Coronavirinae family members (Fig. S1). Protein sequence (FASTA) alignment of SARS-CoV-2 proteins with other coronavirus species from the bat, civet, pangolin, including SARS-CoV-2 were performed using P-BLAST. The analysis showed high sequence similarity for RdRp (> 95%), helicase (> 99%), and main protease (> 95%) proteins (Fig. S1). SARS-CoV-2 spike (S) protein showed maximum sequence homology with pangolin coronavirus (92.43%) and over 76% homology with coronaviruses from other species.

### Mutational landscape of the SARS-CoV-2 genome to detect the emergence of VOCs

**Figure 2:**
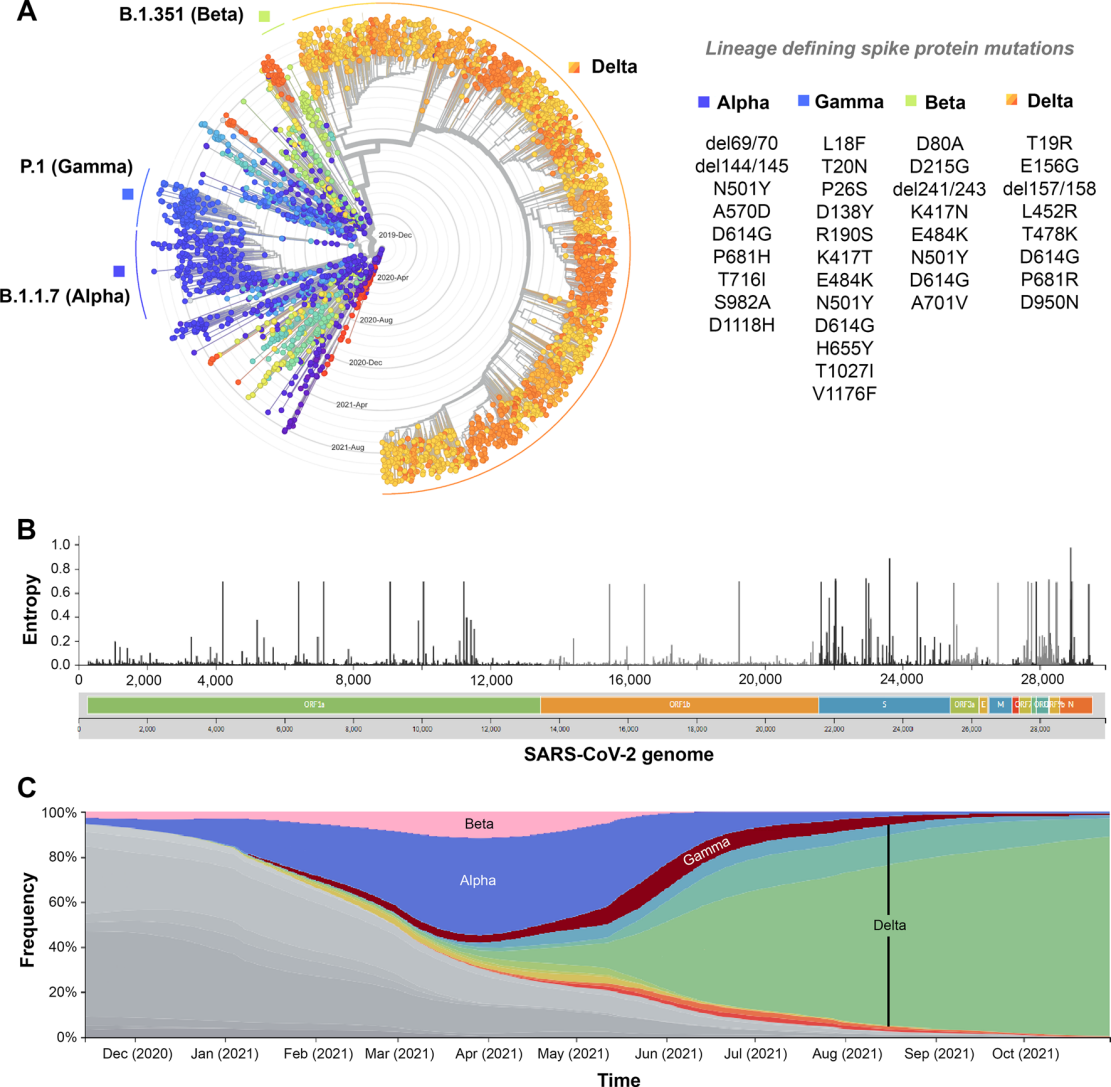
**(A)** Radial phylogenetic time tree based on 3525 SARS-CoV-2 RNA sequences that comprise major lineages of SARS-CoV-2 such as, *Alpha*, *Beta*, *Gamma,* and *Delta*. The radial phylogenetic time tree, with concentric circles showing the dates of sample collection; earlier dates of sample collection are closer to the center. **(B)** Lineage-defining Spike protein mutations for SARS-CoV-2 variant of concerns (VOCs) that are most prevalent worldwide, determined from the SARS-CoV-2 sequences and metadata submitted in GISAID (https://www.gisaid.org/), using the Nextstrain ncov pipeline (https://github.com/nextstrain/ncov). **(C)** Relative frequencies of SARS-CoV-2 VOCs over months. The Delta SARS-CoV-2 lineage outcompeted all other variants.

We have analyzed 5,053,231 SARS-CoV-2 genome sequences deposited to the GISAID database till November 2021. By phylodynamic time tree analysis, we further determined the lineage diversity of SARS-CoV-2 variants over time (Fig. 2A). We have detected nonsynonymous single nucleotide substitutions in 4111 amino acid positions of ~ 30 Kb viral genome. The amino acid diversity as estimated by entropy calculation was measured over the genome (Fig. 2B). Among these mutations, 66.8% belonged to ORF1ab, 12.9% to Spike and 5% belonged to N-protein, and the rest belonged to other viral proteins. Phylodynamic time tree analysis showed 31 amino acid positions [9, 6, and 5 positions of Spike, ORF1a and N-protein and 11 positions of ORF1b, ORF3a, ORF7a/b, ORF8, and ORF9b] on the SARS-CoV-2 genome to possess entropy over 0.5. Most diversity was observed for amino acid position 203 (entropy: 0.97) of the N-protein and 681 (0.89), 452 (0.72), and 157 (0.71) amino acid positions on Spike protein. The spike D614G mutation became predominant (~ 70%) since March 2020^75^, and till December 2020, a few other Spike protein mutations such as P681H, N501Y in the backbone of D614G started gaining high prevalence. Previous studies have already shown the functional impact of spike D614G and N501Y mutations that provided a selective advantage to these mutations in viral entry, either through the interaction of ACE2 or by S1-S2 cleavage^75–78^. Moreover, the N501Y mutation in S protein is present in B.1.1.7 variant (*Alpha*, first reported from the UK), B.1.351 variant (*Beta*, first reported from South African variant), and P.1 variant (*Gamma*, first reported from the BrazilF) and exhibited stronger interaction with the host ACE-2 receptor^49^. During April 2021, after emergence, the Delta variant with characteristic Spike mutations such as T19R, E156G, 157–158 deletion, L452R, T478K, P681R, and D950N within the D614G backbone started outcompeting the other SARS-CoV-2 variants (Fig. 2C). It gradually became the most frequent SARS-CoV-2 lineage worldwide (Fig. S2). The Spike protein’s amino acid positions that possess the highest entropy, i.e., 681, 452, and 157, all of them belong to the Delta variant of SARS-CoV-2.

### Homology modeling, refinement, and structure validation of wild type and mutant S proteins

The template sequence for modeling the wild type and spike mutants was identified by PDB alignment using the BLASTp program. The BLAST search against the PDB database provided homology sequences against several structures. Due to the unavailability of the crystal structure of the region of interest, we have taken EM structure as a template for each different mutation based on query coverage, highest GMQE score, and percent (%) identity. The GMQE score is generated by the target-template alignment of the modeled protein in SWISS-MODEL, and the value is expressed as a number between zero and one. Higher GMQE scores indicate increased structural reliability. The GMQE score for the modeled protein was found to be 0.98, which indicates good model accuracy. We prepared five sequence files by changing the amino acid in the specified region, i.e. wild type (no alteration), and four VOCs, namely *alpha*, *beta*, *gamma*, and *delta* with defined spike mutations (Table 1). These sequences were modeled against the template obtained in SWISS-MODEL based on GMQE and identity (Table 2). After the model refinement, each structure was validated through the generation of the Ramachandran plot using the PROCHECK tool (Table 2). Ramachandran plot confirmed that most of the amino acids of the modeled structures lie in the allowed region (> 90%), which indicates the stability of the structures (Table 2).

### Effect of point mutations on the structure, topology, and stability of the spike protein

Comparative analysis of protein topology and secondary structure between wild type and different variants of spike protein (*alpha, beta, gamma,* and *delta*) was done using PredictProtein and ProtParam. Our analysis confirms no significant structural changes in the spike protein among different variants compared to the wild-type protein. However, local conformational changes had occurred due to the insertion or deletion of post-translational modification sites in the spike mutants (Table 3). The percentages of helices, extended strand, and coiled-coil formation vary between the mutant and wild-type proteins. Interestingly these variations did not cause any significant changes in the solvent accessibility (buried/exposed/intermediate) of the spike protein (Table 3). Other parameters like iso-electric pH and the aliphatic index remained similar between different variants. The instability index shows that all the variants have identical overall stability compared to the wild-type protein (Table 3).

**Table 3:** Effect of the point mutations on the topology, structure and conformation of the wild-type and mutant spike proteins.

**Using PredictProtein server**					
Protein topology	WT	Alpha	Beta	Gamma	Delta
Signal peptide	AA 1–13	AA 1–12	AA 1–12	AA 1–14	AA 1–13
Extracellular	AA 14–874 and 1239–1273	AA 13–869 and 1237–1269	AA 13–871 and 1239–1271	AA 15–874 and 1239–1273	AA 14–872 and 1239–1271
Transmembrane helix	AA 875–888 and 1214–1238	AA 870–882 and 1212–1236	AA 872–888 and 1214–1238	AA 875–888 and 1214–1238	AA 873–885 and 1214–1238
Cytoplasmic	AA 890–1213	AA 883–1211	AA 886–1213	AA 889–1213	AA 886–1213
Disordered region	AA 807–809	AA 803–808	AA 805–809	AA 807–811	AA 805–810
**Secondary structure composition**					
Helix	17.67%	19.15%	18.65%	18.93%	17.94%
Strand	28.28%	26.64%	27.22%	26.32%	25.49%
Others	54.05%	54.22%	54.13%	54.75%	56.57%
**Solvent accessibility**					
Buried	60.09%	60.20%	61.05%	60.33%	60.58%
Exposed	31.34%	30.73%	29.74%	30.40%	30.68%
Intermediate	8.56%	9.06%	9.21%	9.27%	8.73%
**Using ProtParam server**					
**Protein information**					
Isoelectric pI	6.24	6.35	6.64	6.39	6.94
Protein instability index	33.01 (stable)	32.80 (stable)	33.17 (stable)	32.71 (stable)	32.81 (stable)
Aliphatic index	84.67	85.02	84.42	84.45	84.5
Folding rate (fold rate)	The folding rate, ln(kf) = −11/s	The folding rate, ln(kf) = 3.52/sec	The folding rate, ln(kf) = −8.46/s	The folding rate, ln(kf) = −3.9/s	The folding rate, ln(kf) = −7.03/s

**Figure 3:**
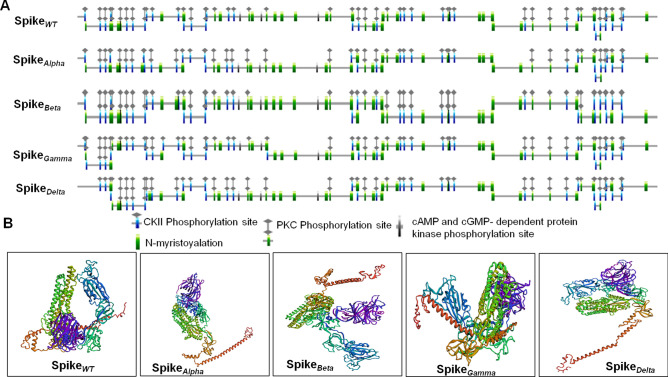
**(A)** Prediction of different post-translational sites in wild-type and mutant spike proteins. **(B)** Prediction of the tertiary structure of wild-type and mutant spike proteins using RoseTTAFold tool.

Although we did not observe any significant structural changes, analysis of motifs on spike protein revealed differential patterns (Fig. 3A, Table 3). We were able to identify 22 N-glycosylation motifs which recognize Asn as a central amino acid in the wild type, *alpha* and *beta* variants, whereas, in *gamma*, two more glycosylation motifs were generated at amino acid residue 20–23 and 188–191 due to the mutations T20N and R190S respectively (Fig. 3A, Table 3). In the *delta* variant, due to the mutation T19R, the glycosylation motif at amino acid residue 17–20 is absent (Fig. 3A, Table 3). Another critical motif, the protein kinase C phosphorylation site, was observed 13 times in wild-type and alpha variants, while in beta variant, a new site has been generated at 1035–1037 (Fig. 3A, Table 3). On the other hand, in the *gamma* and *delta* variants, the sites at 415–417 and 19–21 are absent due to the mutations at K417T (gamma) and T19R(delta), respectively. The phosphorylation motif casein kinase II (CK2) is present 14 times in the wild, beta, and delta variants, while in alpha, one site is absent at 982–985 due to the mutation S982A. In the gamma variant, one additional site is generated at 417–420 due to mutation K417T (Fig. 3A, Table 3). Two other vital motifs were also identified in spike proteins that were present in wild-type as well as in all the variants. These are the cAMP-cGMP-dependent protein phosphorylation site and cell attachment site at the RBD potion of spike protein (Fig. 3A, Table 3). Apart from the different phosphorylation sites, the N-Myristylation site, which acts as an essential post-translational modification, was also identified in spike protein. In wild-type protein 25, such N-Myristylation sites were identified, while in the alpha variant, two additional sites were evolved at residues 140–145 and 610–615 due to mutations (Fig. 3A, Table 3). In the beta variant, three more sites were evolved at residues 215–220, 411–416, and 610–615, while due to the mutation A701V, the site between 700–705 is absent (Fig. 3A, Table 3). In gamma and delta variants, the site at 610–615 is evolved, while in gamma, another new site at 210–215, and in the delta at 140–145 has evolved due to different mutations (Fig. 3A, Table 3).

Changes in protein-folding rate in wild-type and mutant proteins were observed by using FOLD-RATE software. The protein-folding rate is usually inversely proportional to the time that a given protein needs to collapse into its stable tertiary structure. We observed that wild-type spike protein has a fast-folding rate (ln Kf −11 s^−1^), while for the alpha mutant, it becomes 3.52 s^−1^ due to the mutations. On the other hand, beta, gamma, and delta variants have ln Kfvalues of −8.46, −3.9, and −7.03 s^−1^, respectively (Table 3).

We further determined the structural changes in the wild-type and mutant spike proteins using the structure-prediction tool RoseTTAFold^59^. The Structural comparison between wild-type and different variants of spike proteins revealed a striking difference in their tertiary structure (Fig. 3B). The closed-loop structure in wild-type spike protein is not present in the variants. In *alpha*, *beta*, and *delta* variants, the C-terminal portion appeared like a protruding hanging end (orange color), whereas in *gamma,* it is folded in between the rest of the structure (Fig. 3B). The beta and gamma variants also formed a groove between the N-terminal domain and RBD (Purple and blue color, respectively), which is absent in *alpha* and *delta* variants (Fig. 3B). Thus the results indicated that the mutations in the spike protein had induced substantial conformational change in the spike protein.

### Molecular docking of the target proteins with statins

#### RNA dependent RNA polymerase (RdRp)

The cryo-EM structure of SARS-CoV-2 RdRp bound to the drug remdesivir at nsp12 was first deduced by Yin. et al. in 2020^79^, where they reported the existence of the complex either in the apo form or in a complex with the primer RNA (PDB ID: 7BV2). The structural features of RdRp revealed that the polymerase domain consisted of a ‘finger’ domain (amino acid residues: 398–581, 628–687), a ‘palm’ domain (amino acid residues: 582–627, 688–815), and a ‘thumb’ domain (amino acid residues: 816–919) and also an additional nidovirus-unique N-terminal extension (amino acid residues: 1–397)^80^. As per the PDBsum record, 7BV2 has seven beta-sheets, forty-six α-helices, fifty-eight beta-turns, and two gamma-turns.7BV2 comprises six active sites such as AC1, AC2, AC3, AC4, AC5, and AC6, respectively, of which AC3 and AC6 constitute the remdesivir binding site.

**Figure 4:**
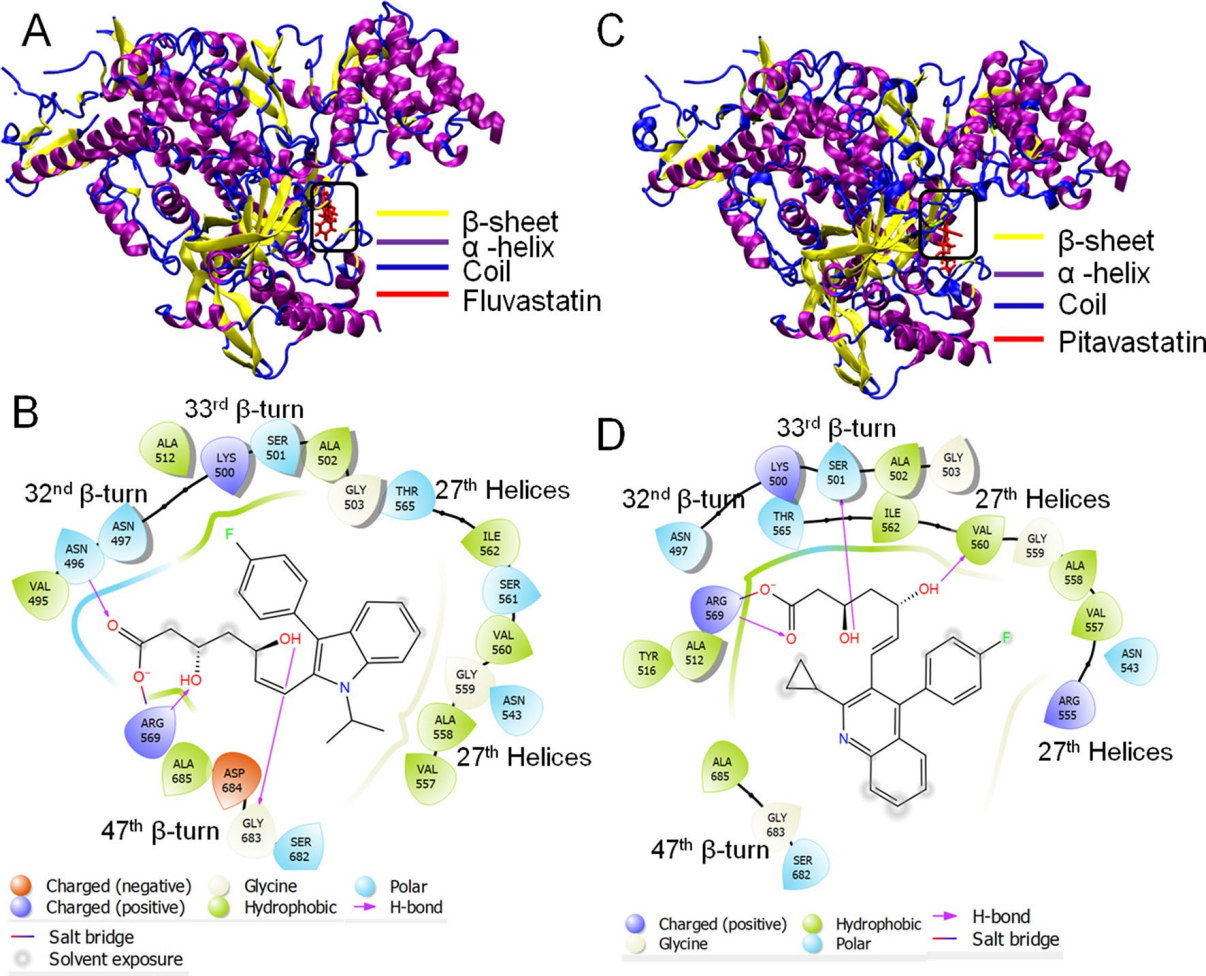
Molecular docking study of fluvastatin and pitavastatin to RdRp. **(A,C)** 3D diagram of fluvastatin-RdRp complex and pitavastatin-RdRp complex, respectively. **(B,D)** 2D diagram for representation of interactions of ligand molecule with the amino acid residues at the binding site; **(B)** fluvastatin-RdRp complex and **(D)** pitavastatin-RdRp complex. All 3D images were generated using the VMD (visual molecular dynamics) software (version 1.9.3) and 2D images were generated by the graphical user interface Maestro, Schrödinger (https://www.schrodinger.com/products/maestro).

Molecular docking of 7BV2 with the statin molecules revealed that only fluvastatin, pitavastatin, pravastatin, rosuvastatin, and simvastatin qualified with a docking score while the other statin molecules did not qualify the screening (Table 4). Among those statins, fluvastatin and pitavastatin were the best candidate molecules with higher binding affinities (Fig. 4, Figs. S3 and S4, Table 4). In contrast, the other statin molecules did not exhibit strong binding (Table 4). The 3D structures of Rdp-ligand complexes and their binding sites are shown in Fig. 3 and Figs. S3 and S4(A,B). Both the molecules bind at the remdesivir binding site of the enzyme. The glide score, emodel score,and energy values for fluvastatin were −7.44, −60.23, and −57.076 kcal/mol(Table 4), and the corresponding values for pitavastatin were −7.5, −62.341, and −58.654 (kcal/mol),respectively (Table 4).The MMGBSA score for fluvastatin and pitavastatin were −32.13 and −49.96 kcal/mol, respectively. In our study, we observed that the glide score, emodel score, glide energy, and MMGBSA scores for remdesivir (positive control) were −7.312, −56.672, −71.38, and −33.38 kcal/mol, respectively (Table 4).

**Table 4:** Docking analysis of the statins against target proteins of SARS-CoV2, except the spike protein.

target protein	PDB ID	Selected ligands	Glide score	Glide energy (kcal/mol)	Glide emodel (kcal/mol)	MMGBSA (kcal/mol)	Centre of binding location		
							X	Y	Z
RdRp	7BV2	Fluvastatin	−7.44	−57.08	−60.23	−32.13	84.51	100.41	109.24
		Pitavastatin	−7.5	−58.65	−62.34	−49.96	85.11	100.70	109.73
		Pravastatin	−6.03	−38.82	−47.42	−12.98	–	–	–
		Rosuvastatin	−5.93	−39.29	−44.43	−23.38	–	–	–
		Simvastatin	−4.59	−27.09	−29.21	−27.84	–	–	–
		Remdesivir	−8.0	−44.84	−54.61	−33.38	98.54	87.45	101.57
3CL-Pro	6LU7	Fluvastatin	−7.34	−48.62	−61.75	−43.54	−10.37	15.56	68.24
		Pitavastatin	−7.12	−45.78	−57.56	−31.67	−11.48	13.49	69.05
		Cerivastatin	−5.2	−36.36	−46.71	−28.20	–	–	–
		Atorvastatin	−4.9	−50.73	−58.99	−48.21	–	–	–
		Rosuvastatin	−4.1	−29.65	−34.64	−21.80	–	–	–
		Simvastatin	−3.5	−26.18	−27.54	−34.47	–	–	–
		Ml188	−6.38	−47.05	−67.01	−39.93	−9.39	16.16	68.62
Helicase	6ZSL	Fluvastatin	−11.33	−58.72	−66.51	−34.70	−20.68	33.63	−25.84
		Pravastatin	−7.61	−48.41	−55.67	−33.00	−10.10	28.20	−51.15
		Atorvastatin	−6.0	−35.89	−46.18	−44.46	–	–	–
		Pitavastatin	−6.0	−39.26	−56.94	−29.26	–	–	–
		Cerivastatin	−5.7	−40.33	−52.88	−33.34	–	–	–
		Rosuvastatin	−4.8	−32.94	−33.07	−35.89	–	–	–
		Lumacaftor	−7.18	−44.03	−59.88	−29.02	−20.89	31.41	−26.40

The binding pockets for fluvastatin and pitavastatin were further analyzed using the Discovery Studio client. The fluvastatin binding pocket was found to be hydrophilic in nature (Fig. S3C) due to the presence of hydrophilic amino acids like ASN 496, ARG 569, ASN 497, SER 501, THR 565, ASN 543, and SER 561. Since, except arginine, all other hydrophilic amino acids are uncharged, the binding pocket is nearly neutral (Fig. S3D). The presence of both the H-bond donor residues (ASN 496 and ARG 569) and H-bond acceptor residues (GLY 683)has made this site favorable for the formation of H-bond with the ligand (fluvastatin). These residues, present in 32nd and 47th β-turn, formed conventional H-bonds with the carboxyl and hydroxyl groups of fluvastatin (Fig. 4B). Moreover, the alkyl group of fluvastatin had hydrophobic interactions with the VAL 557 residue(Fig. 4B). All these interactions are favorable for forming a stable fluvastatin-RdRP complex.

The calculated LogP and pKavalues of fluvastatin and pitavastatin are similar (LogP: 4.85 and 4.8; pKa: 4.5 and 4.3) (Table S1). Hence the binding pocket for pitavastatin showed similar properties to the fluvastatin-binding pocket. The presence of uncharged polar amino acids like SER 501, ASN 543, ASN 497, THR 565, and positively charged basic amino acids like ARG 569, LYS 500, and ARG 555contribute to the hydrophilicity of the pitavastatin binding pocket. In contrast, the presence of uncharged nonpolar amino acids like VAL 557, ILE 562, VAL 560, VAL 557, ALA 512, ALA 558, and ALA 502 contribute to the hydrophobicity of the binding pocket (Fig. S4C). The presence of basic amino acids imparts a slight basic character to the binding pocket of pitavastatin to RdRp (Fig. S4D). The carboxylic acid group and hydroxyl groups of pitavastatin form H-bonds with the β-turn (32nd and 33rd) and 27th helical region of the protein. SER 501 and VAL 560 act as H-bond acceptors, whereas ARG 569 is H-bond act as the donor (Fig. 4D). The negatively charged O-atom (formed due to ionization of – COOH group) formed salt-bridge with ARG 569. The π-electron cloud of the fluorophenyl group interacted with alkyl groups (–CH_3_) of VAL 557, located in the 14th β strand of D sheet, and formed π-alkyl bond. The electron cloud of the quinoline ring contributed to interaction with GLY 683 residue, located in the 35th β strand of the D sheet. All these interactions contribute to a stable interaction of pitavastatin with RdRp.

Thus the results indicate that both Fluvastatin and Pitavastatin have a strong binding affinity towards RdRP and bind at the remdesivir binding pocket of the enzyme, and these two statins may inhibit enzyme activity.

#### 3CL-Pro or M-protease

As per the PDBsum record, 3CL-Pro (306 amino acid long, PDB ID: 6LU7) has a single chain consisting of two beta-sheets, ten helices, twenty-six beta-turns, and three gamma turns. A previous report by Jin. et al. (2020) demonstrated that the enzyme consists of an active site to which the inhibitors can bind and inhibit the enzyme activity^81^. The inhibition site comprised of the amino acids such as HIS 41, MET 49, TYR 54, PHE140, LEU 141, ASN 142, GLU 166, HIS 163, MET 165, MET 165, LEU 167, HIS 172, PHE 185, and GLN 192 and the natural compounds lime Ebselen, Disulfiram, Tideglusib, Carmofur, Shikonin and PX-12 were reported to bind at that site^81^. Recently, it was reported that pitavastatin and fluvastatin could bind to 3-CL-Pro with the binding energy of – 8.2 and – 7.7 kcal/mol^82^, but the report lacks detailed docking analysis and dynamics studies to support the prediction.

**Figure 5:**
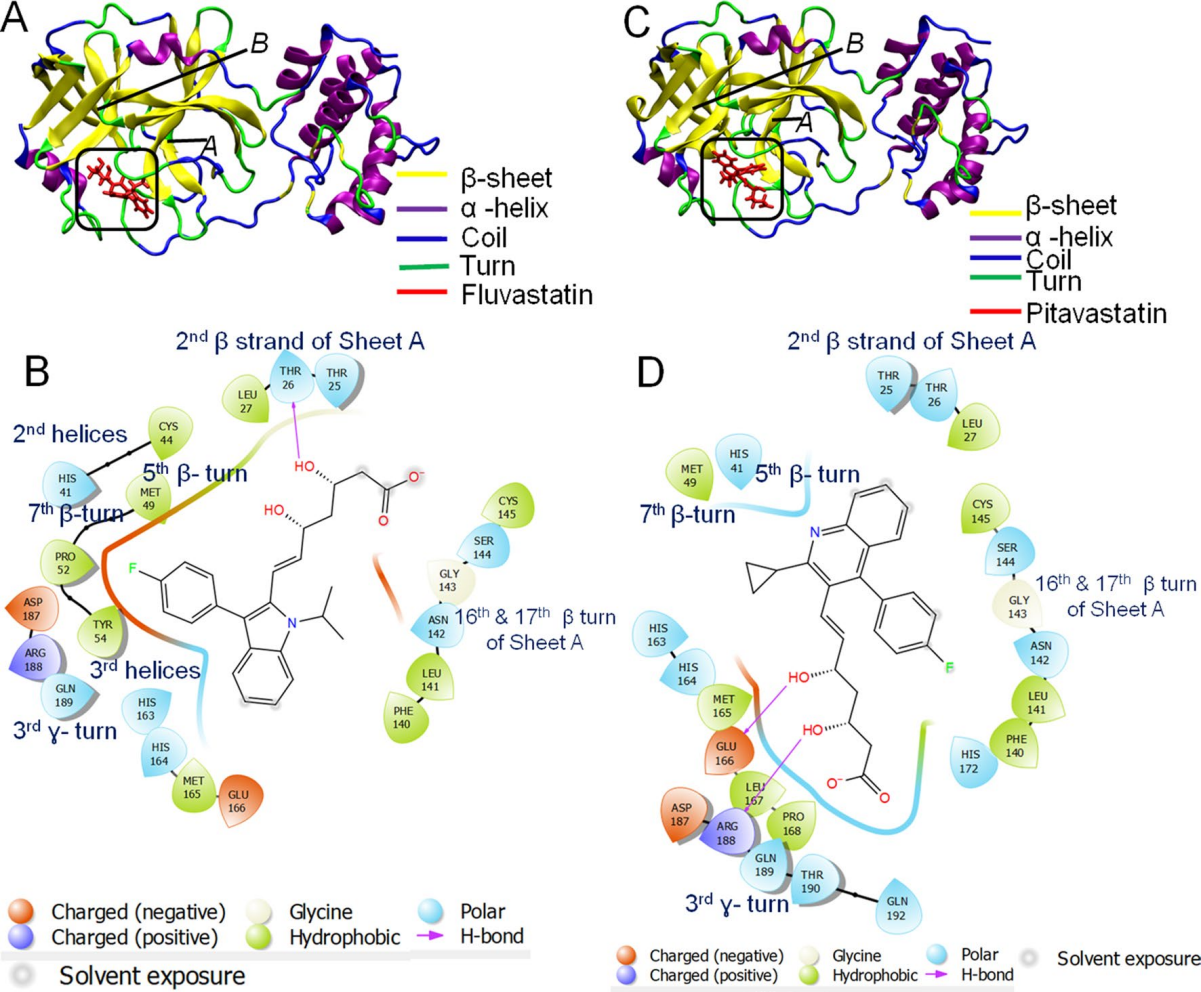
Molecular docking study of Fluvastatin and pitavastatin to 3-CL-Pro. **(A,C)** 3D diagram of fluvastatin-3-CL-Pro complex and pitavastatin-3-CL-Pro complex, respectively. **(B,D)** 2D diagram for representation of interactions of ligand molecule with the amino acid residues at the binding site; **(B)** fluvastatin-RdRp complex and **(D)** pitavastatin-RdRp complex. All 3D images were generated using the VMD (visual molecular dynamics) software (version 1.9.3) and 2D images were generated by the graphical user interface Maestro, Schrödinger (https://www.schrodinger.com/products/maestro).

Molecular docking of the statins with 6LU7 revealed that Fluvastatin and pitavastatin are the best ligands to bind at the active site (site of inhibition). In contrast, the other statin molecules did not exhibit strong binding(Table 4, Fig. 5). The detailed analysis of ligand–protein complexes is represented in Fig. 5A,B and Fig. S5 (for fluvastatin) and Fig. 5C,D and Fig. S6 (for pitavastatin). The values of glide score, emodel, and glide energy for Fluvastatin were −7.338, −61.748, and −48.617 (kcal/mol), respectively, whereas the same for pitavastatin were −7.119, −57.563, and −45.785(kcal/mol), respectively (Table 4). The MMGBSA score for fluvastatin and pitavastatin were −43.54 and −31.67 kcal/mol, respectively. In our study, we observed that the glide score, emodel score, glide energy, and MMGBSA scores for ML188 (positive control) were −6.38–47.05–67.01, and −39.93 kcal/mol, respectively (Table 4). Thus the results indicated the strong affinity of fluvastatin and pitavastatin towards the enzyme.

Characterization of the binding cavity of Fluvastatin revealed that it is partly hydrophilic (Fig. S5C, Blue color). The binding cavity is comprised of the residues like THR 25, THR 26, TYR 54, ASP 187, and GLN 189, which impart a polar (hydrophilic) character to the binding pocket(Fig. S5C). As per ionizibility, the binding site mainly was neutral due to the presence of the residues like THR 26, HIS 41, TYR 54, and CYS 145 (fade white color, Fig. S5D), while only GLU 166 was acidic (slightly brown in color, Fig. S5D). The presence of H-donor amino acid residues (THR 26, HIS 41, TYR 54, and CYS 145) and H-acceptor amino acid residue (GLU 166) results in H-bond formation with fluvastatin. Additionally, the fluorine atom of fluorophenyl (C_6_H_5_F-) group of fluvastatin was involved in chemical interaction with ASP 187 residue, present in 2nd ɣ-turn of the protein, while the O-atoms of polyhydroxy groups (-OH) interacts with the HIS 41 residue, present in 2nd helices. Moreover, the alkyl group of fluvastatin had hydrophobic interactions with CYS 145 and HIS 163, present on the 12th β strand of β sheet and 17th β turn, respectively (Fig. 5B). All these interactions contributed to a stable fluvastatin-3CL-Pro complex.

Due to the similar LogP values (Table S1), the binding pocket for pitavastatin is similar to that of fluvastatin. The binding site of pitavastatin was slightly basic due to the occurrence of the basic amino acid residues like HIS 41, HIS 163, HIS 164, HIS 141, and ARG 188(Fig. S6D). HIS 41 acts as H-donor while GLU 166 and ARG 188 act asH-acceptor and participate in the formation of conventional H-bond with hydroxyl groups of pitavastatin(Fig. S6E). The electron cloud of the fluorophenyl group of pitavastatin contributed to π–π stacked interaction with LEU 141, present in the 16th β turn of the protein (Fig. 5D). All these interactions contribute to the formation of a stable pitavastatin—3CL-Pro complex. The compound ML188 was taken as the positive control^83^ (Table 4).

#### Helicase

Helicase (Nsp13) is a multi-functional protein that consists of the N-terminal Zn-binding domain (ZBD) and the helicase domain (Hel). The N-terminal region consists of twenty-six cysteine residues that form the Zn^2+^ binding domain, whereas the helicase domain comprises a conserved motif at the C-terminus^84^. Helicase requires ATP hydrolysis for its action, and the residues such as SER 310, LYS 288, and GLU 375 constitute the ATP-binding site or the active site^85^. The crystal structure of the SARS-CoV-2 helicase (resolution 1.94 Å) was reported by Newman et al. (PDB ID: 6ZSL) (https://www.rcsb.org/structure/6ZSL). As per the PDBsum record, helicase consists of nine beta-sheets, thirty-four beta-strands, twenty-three helices, forty-four beta turns, and three gamma-turns.

Molecular docking of 6ZSL with the statins revealed that fluvastatin exhibited the strongest binding affinity with the glide score, emodel, and glide energy values of −11.333, −66.511, and −58.72 (kcal/mol), respectively (Table 4). The MMGBSA score for fluvastatin was −34.7 kcal/mol. In our study, we observed that the glide score, emodel score, glide energy, and MMGBSA scores for Lumacaftor (positive control) were −7.18, −44.03, −59.88, and −29.02 kcal/mol, respectively (Table 4). Thus the results indicated the strong affinity of fluvastatin towards the helicase.

**Figure 6:**
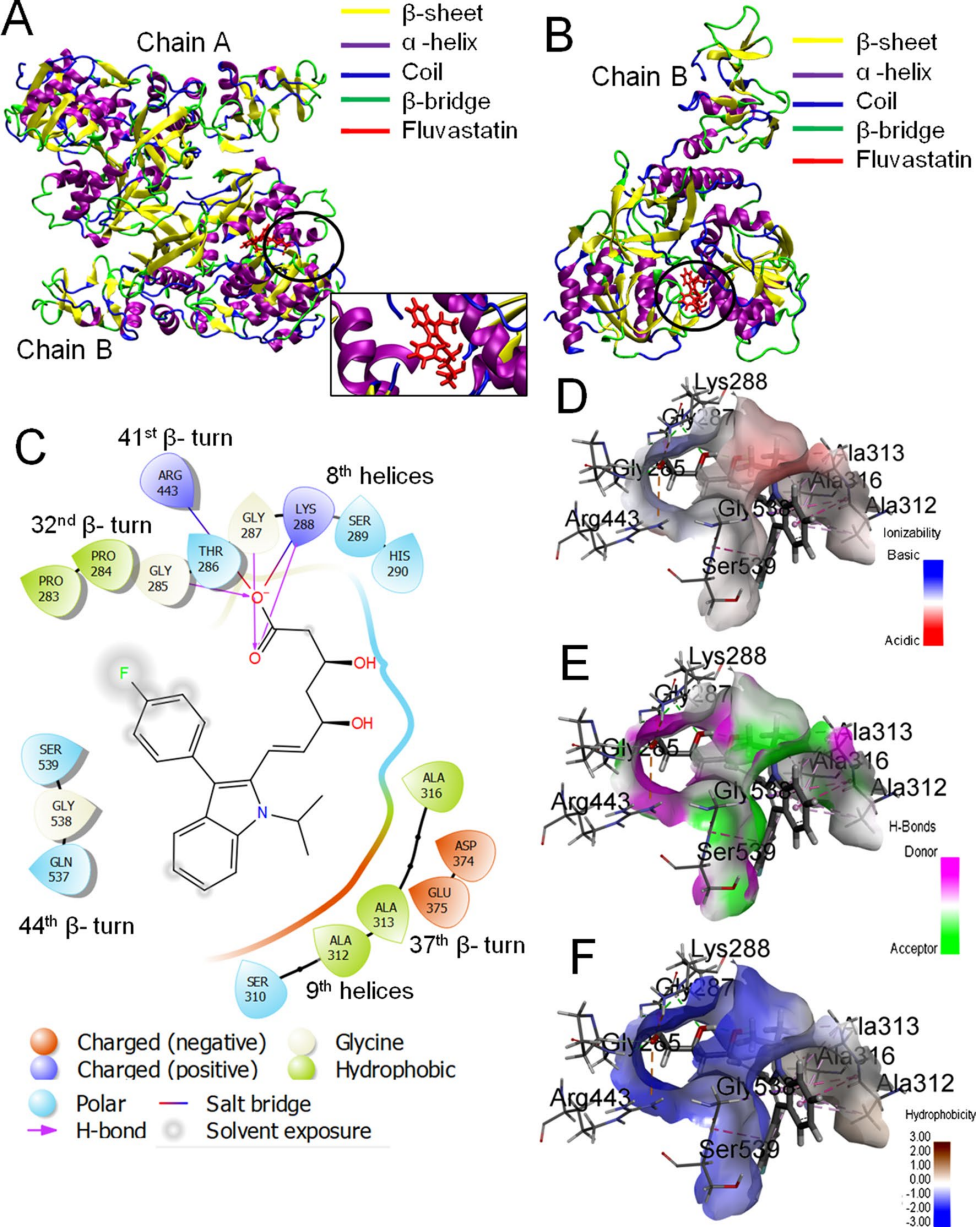
Molecular docking study of fluvastatin to helicase. **(A)** 3D diagram of ligand–protein complex (chain A and Chain B). **(B)** 3D diagram of Fluvastatin bound helicase complex (chain B only). **(C)** 2D diagram for representation of interactions of Fluvastatin with the amino acid residues at the binding site. **(D–F)** Mapping of binding site cavity according to ionizability, H-bond donor–acceptor residue, and hydrophobicity, respectively, using Discovery Studio Visualizer software, v21.1.0.20298 (https://discover.3ds.com/discovery-studio-visualizer-download). All 3D images were generated using the VMD (Visual Molecular Dynamics) software (version 1.9.3) and 2D images were generated by the graphical user interface Maestro, Schrödinger (https://www.schrodinger.com/products/maestro).

The 3D structure of the fluvastatin-helicase complex is shown in Fig. 6A,B, while the major 2D interactions are shown in Fig. 6C. Further characterization of the fluvastatin-binding site on helicase revealed that the hydrophilic residues (GLY 265, THR 286, GLY 287, LYS 288, HIS 290, SER 310, ARG 443, GLN 527, ASP 374, GLU 375, and SER 539) outnumbered the hydrophobic residues (PRO 283, PRO 284, ALA 312, ALA 313, and ALA 316), to make the pocket preferentially hydrophilic (Fig. 6D–F). The pH profile of the binding cavity indicated that the region having ASP 374 and GLU 375 residues is acidic, whereas that having ARG 443, LYS 288, and HIS 290 are basic (Fig. 6D). The ionized form (negatively charged) of fluvastatin was in proper pose to form two salt bridges between the –COO^−^ group and basic amino acids LYS 288 of 8th helices, ARG 443of 41st β-turn. The binding cavity is rich with both the H-bond donor and acceptor residues which facilitate the formation of multiple H-bonds with Fluvastatin (Fig. 6E). The negatively charged O-atom of −COO^−^ group of Fluvastatin formed H-bond with GLY 285, whereas the carbonyl O-atom formed H-bond with GLY 287, and LYS 288. The LYS 288 residue formed an additional H-bond with the −OH group attached to the aliphatic chain. Moreover, ten hydrophobic interactions, such as alkyl-alkyl, pi-alkyl, pi-sigma, amide-pi stacked, were observed between the π electron-rich aromatic rings of fluvastatin and the surrounding residues like GLY 538, ALA 312, ALA 313, and ALA 316. All these interactions had synergistic effects to make helicase the preferable target protein for fluvastatin. Interestingly, the previous study had revealed that the ATP binding site of helicase is comprised of amino acid residues like K288, E375, Q404, R443, and R567^36^. Our study observed that fluvastatin binds to helicase in a similar region, and the residues like LYS 288 and ARG 443 play important roles in this interaction (Fig. 6B and Fig. S7). Hence it may be concluded that fluvastatin may interfere with the ATP binding site of helicase and inhibit the enzyme's activity.

#### Wild type and mutant spike proteins from VOCs

Spike protein is one of the essential proteins required for the entry of SARS-CoV2 into the host cell. The viral entry is mediated by the interaction of the RBD (receptor binding domain) of the S1 subunit of spike with the ACE2 receptor of the host cell membranes. The structural insights of the different conformational states of the S-protein and S1(RBD)-ACE2 complex have been reported^86^. Further S1- subunit is composed of four domains, namely the N-terminal domain (NTD), RBD, and two C-terminal domains (CTDs), CTD-1, and CTD-2^87^. In our modeled S-proteins (wild type S protein and the mutants), the amino acids 14 to 685 constitute the S1 region, while the residues 686 to 1273 comprise the S2 site, which corroborates with the published report^88^. As mentioned earlier, new lineages of SARS-CoV-2 had emerged with higher transmissibility and immune-escape properties, which are known as the VOCs. The signature mutations of the SARS‐CoV‐2 VOCs such as B.1.1.7 (alpha), B.1.351 (beta), P.1(gamma), and B.1.617.2 (delta) are enlisted in Table 1. The concerned mutations for each VOC were introduced in the wild-type S-protein, and the mutant S-proteins were modeled accordingly. We performed blind docking of all the nine statin molecules against the wild-type and mutant S-proteins, using the Schrödinger suite.

##### Wild type spike protein (Spike_*wild-type*_)

**Figure 7:**
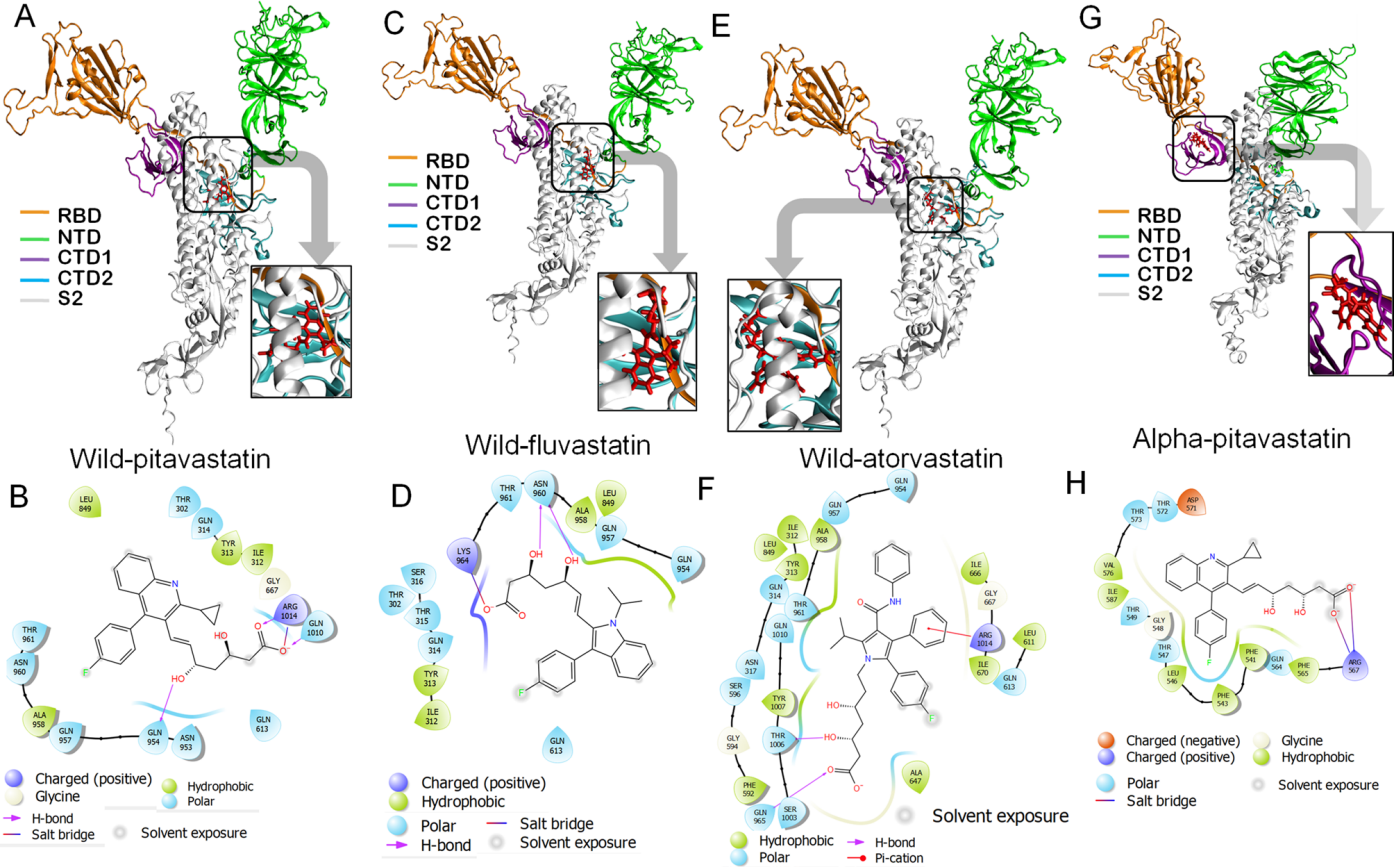
Molecular docking study of pitavastatin, Fluvastatin, and atorvastatin to Spike_*WT*_ and Spike_*Alpha,*_ respectively. **(A,C,E)** 3D diagram of pitavastatin Spike_*WT*_ complex, fluvastatin Spike_*WT*_ complex, and atorvastatin Spike_*WT*_ complex, respectively. **(B,D,F)** 2D diagram for representation of interactions of pitavastatin, Fluvastatin, and atorvastatin molecules with the amino acid residues at the binding site of Spike_*WT*_. **(G,H)** 3D diagram of pitavastatin–Spike_*Alpha*_ complex and 2D diagram for representation of interactions of pitavastatin, Fluvastatin, and atorvastatin molecules with the amino acid residues at the binding site of Spike_*Alpha.*_ All 3D images were generated using the VMD (visual molecular dynamics) software (version 1.9.3) and 2D images were generated by the graphical user interface Maestro, Schrödinger (https://www.schrodinger.com/products/maestro).

Molecular docking of all statin molecules against Spike_*wild-type*_ protein reveals that pitavastatin has the strongest binding affinity for the target protein, with a docking score of −8.9 kcal/mol, followed by fluvastatin and atorvastatin (docking scores −8.2 and −8 kcal/mol, respectively) (Table 5). We observed that all the three statins bind at the hind region (joining site of NTD and RBD site) of Spike_*wild-type*_ protein in a similar fashion (Fig. 7A–F). The docking score, glide energy, and MMGBSA score of the pitavastatin-SpikeWild complex are −8.937, −48.589, and −52.37 kcal/mol, respectively (Table 5, Fig. 7A,B).The binding site analysis of the pitavastatin- Spike_*wild-type*_ complex revealed that the carboxyl (C=O) and carbonyl (C–O) groups of pitavastatin interact with the positively charged amino acid ARG 1014 to form electrostatic salt-bridge interaction. The hydroxyl group (OH−) of the pitavastatin interacts with GLN 954 and 1010 amino acids resulting in the formation of H-bond. Further, The π-electrons of the fluorophenyl group (C_6_H_5_F-) and tri-cyclic ring of pitavastatin interact with the hydrophobic amino acid residues (ALA 958 and TYR 313) via π-alkyl interactions. The π-amide interaction occurs between the π-electrons of the fluorophenyl group (C6H5F-) and GLN 957 amino acid (Fig. 7B).

**Table 5:** Docking analysis of the statins against wild-type and mutant Spike protein.

Target protein	Selected ligands	Glide score	Glide energy (kcal/mol)	Glide emodel (kcal/mol)	MMGBSA (kcal/mol)
Spike_*Wild*_	Pitavastatin	−8.95	−48.59	−65.88	−52.37
	Fluvastatin	−8.20	−46.84	−62.40	−29.07
	Atorvastatin	−8.02	−61.81	−85.90	−34.76
	Cerivastatin	−6.84	−48.53	−61.22	−36.05
	Pravastatin	−6.62	−42.40	−57.70	−21.28
	Rosuavastain	−5.67	−36.06	−44.06	−36.32
	Mevastatin	−3.20	−28.83	−31.61	−25.16
Spike_*Alpha*_	Pitavastatin	−7.57	−38.72	−53.11	−42.64
	Atorvastatin	−7.14	−59.96	−89.00	−26.67
	Fluvastatin	−6.95	−33.45	−48.14	−41.13
	Pravastatin	−6.69	−40.94	−47.89	−48.29
	Rosuavastatin	−6.45	−44.28	−62.72	−9.27
	Cerivastatin	−4.50	−37.26	−42.73	−22.33
Spike_*Beta*_	Atorvastatin	−7.46	−51.02	−70.21	−45.35
	Cerivastatin	−6.82	−44.34	−56.99	−36.07
	Pravastatin	−6.54	−33.02	−33.71	−25.55
	Pitavastatin	−6.29	−42.35	−60.33	−21.45
	Fluvastatin	−6.05	−38.58	−57.97	−14.97
	Rosuavastatin	−5.32	−40.94	−52.97	−33.86
	Lovastatin	−5.29	−42.11	52.07	−49.00
	Mevastatin	−4.95	−42.25	−52.97	−50.49
Spike_*Delta*_	Fluvastatin	−10.76	−43.71	−50.93	−26.84
	Rosuavastatin	−9.26	−53.41	−62.58	−33.21
	Pitavastatin	−7.19	−47.05	−61.88	−36.70
Spike_*Gamma*_	Pitavastatin	−9.58	−44.46	−60.18	−47.18
	Rosuavastatin	−9.23	−36.86	−45.57	−42.22
	Fluvastatin	−8.37	−35.23	−48.50	−33.99
	Atorvastatin	−7.88	−49.44	−73.26	−55.21
	Pravastatin	−6.26	−39.52	−45.76	−37.07
	Simvastatin	−5.86	−42.85	−56.28	−48.99
	Cerivastatin	−5.78	−31.22	−44.49	−27.78
	Lovastatin	−3.75	−36.56	−41.60	−37.12

The docking score, glide energy, and MMGBSA score of the fluvastatin−Spike_*wild−type*_ complex are −8.194, −46.84, and −29.07 kcal/mol, respectively (Table 5, Fig. 7C,D). It was observed that the carbonyl group (C–O) of fluvastatin interacts with LYS 964 and ASN 960 residues to form salt-bridge and H bonds, respectively. Further, the π-electrons of the indole and fluorophenyl groups interact with ILE 312 and ALA 958 amino acid residues, respectively. In addition, hydrophobic interaction (π-alkyl) occurred between the C-atom of the methyl group (-CH3) of fluvastatin and the benzene ring of TYR 313 amino acid residue (Fig. 7D).

Similarly, atorvastatin was bound at the hind region (joining site of NTD and RBD site) in Spike_*wild-type*_ protein with docking score, glide energy, and MMGBSA scores of −8.02, −61.81, and −34.76 kcal/mol, respectively (Table 5, Fig. 7E,F). The morphological analysis of the binding site of the atorvastatin-Spike_*wild-type*_ complex reveals the following major interactions stabilizing the complex. The carboxyl group (C=O) of atorvastatin forms H-bonding with GLN 965, the hydroxyl groups (−OH) form the same with GLN 314 and THR 1006, while one hydrophobic (alkyl) bond was formed between the methyl (−CH3) group and ALA 958 residue. Moreover, The π-electrons of the phenyl group (C6H5−) interact with ILE 312 to form π-alkyl interactions and GLN 314 to form π-donor H bond. The GLN 613 residue also interacts with two H atoms of atorvastatin to form two carbon-hydrogen (C–H) bonds(Fig. 7F).

##### Spike_*Alpha*_ protein

Molecular docking of all statins against the Spike_Alpha_ protein of SARS-CoV-2 indicates that pitavastatin has the strongest binding affinity for the target protein (Fig. 7G,H, Table 5). The docking score, glide energy, and MMGBSA of pitavastatin against SpikeAlpha protein is −7.56, −38.716, and −42.64 kcal/mol, respectively. The morphological study of the binding site cavity of pitavastatin indicated the following interactions with the target protein (Fig. 7H). The ARG 567 was found to interact with the carboxyl group (C=O), and the hydroxyl group (-OH)of pitavastatin to form an electrostatic salt-bridge and a conventional H-bond, respectively. The F-atom of the fluorophenyl group (C6H5F–) forms an H-bond with the GLN 564 residue. Further, the π-electron cloud of the quinoline ring interacts with THR 549 to form a π-donor H-bond, while the π-electron cloud of fluorophenyl group (C6H5F–) interacts with the alkyl group (CH_3_–) of VAL 576 amino acid residue to form π-alkyl interaction. We further observed that the binding site and location of pitavastatin were also altered in Spike Alpha, compared to the wild-type S protein. For the pitavastatin-Spike_*Wild*_complex, the binding cavity comprises the residues such as THR302, GLN314, TYR313, ILE312, GLY667, ASN953, GLN954, GLN957, ALA958, ASN 960, THR961, GLN613, LEU849, GLN 1010, and ARG1014 residues (Fig. 7B). However, for pitavastatin -Spike_*Alpha-*_ complex, the binding site is comprised of the residues such as PHE 541, PHE 543, LEU 546, THR547, GLY548, THR549, GLN564, PHE565, ARG567, ASP571, THR572, THR573, VAL576, and ILE587 residues (Fig. 7H). Also, pitavastatin was found to bind at the CTD-1 (C-terminal Domain-1) region of Spike_*Alpha*_protein, whereas it was found attached at the hind-region of the Spike_*Wild*_ protein. This difference in the binding pattern of pitavastatin to mutant and wild-type S-protein may be due to the conformational change of the protein structure induced by the defined mutations.

##### Spike_*Beta*_ protein

**Figure 8:**
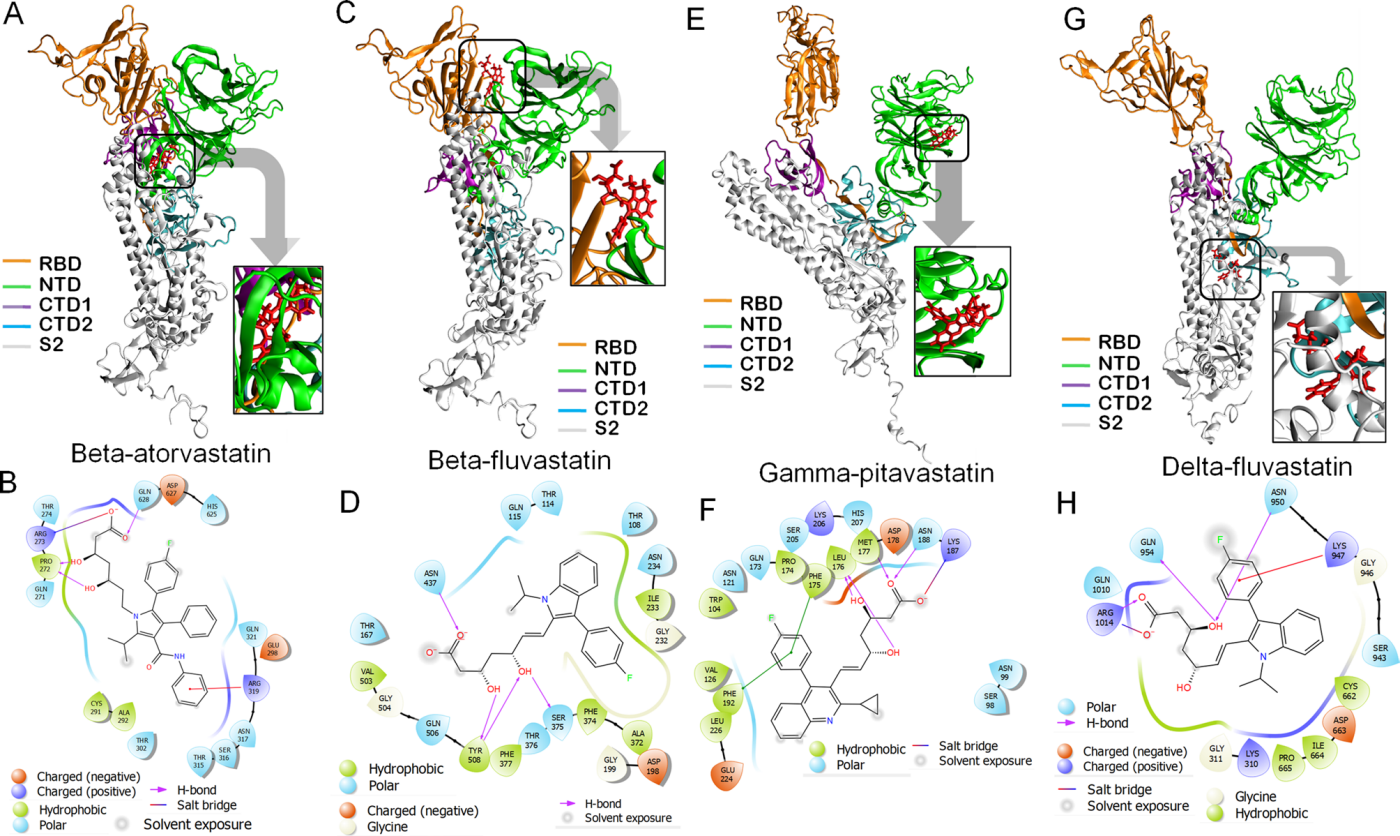
Molecular docking study of atorvastatin and Fluvastatin to Spike_*Beta*_ **(A–D)** and pitavastatin and fluvastatin to Spike_*Delta*_ **(E–H)**, respectively. **(A,C)** 3D diagram of atorvastatin-Spike_*Beta*_ complex, fluvastatin Spike_*Beta*_ complex, respectively. **(B,D)** 2D diagram for representation of interactions of atorvastatin and fluvastatin molecules with the amino acid residues at the binding site of Spike_*Beta,*_ respectively. **(E,F)** 3D diagram of pitavastatin- Spike_*Gamma*_ complex and a 2D diagram for representation of interactions of pitavastatin, Fluvastatin, and atorvastatin molecules with the amino acid residues at the binding site of Spike_*Gamma.*_ , respectively. **(G,H)** 3D diagram of pitavastatin- Spike_*Delta*_ complex and 2D diagram for representation of interactions of pitavastatin, Fluvastatin, and atorvastatin molecules with the amino acid residues at the binding site of Spike_*Delta.*_ , respectively. All 3D images were generated using the VMD (visual molecular dynamics) software (version 1.9.3) and 2D images were generated by the graphical user interface Maestro, Schrödinger (https://www.schrodinger.com/products/maestro).

Molecular docking of the statin molecules against Spike_*Beta*_protein reveals that atorvastatin has the strongest binding affinity for the target protein (Fig. 8A,B, Table 5). The docking score, glide energy, and MMGBSA of atorvastatin against Spike_*Beta*_ protein are −7.454, −51.024, and −45.35, respectively (Table 5). Binding site analysis of the atorvastatin-Spike_*Beta*_ complex shows that the carbonyl group (C–O), carboxyl group (C=O), and the hydroxyl group (–OH) of atorvasta_5_tin interacts with ARG 273 to from a salt-bridge, ARG 319 and GLN 628 to form H-bonds, and PRO 272 to create another H-bond, respectively. Also, the F-atom of the fluorophenyl group (C_6_H_5_F–) interacts with ASP 627 amino acid residue to form halogen interaction. The π-electrons of the fluorophenyl group (C_6_H_5_F–), pyrrole ring, and phenyl ring (C_6_H–) interact with ALA 292 amino acid to form π-alkyl interactions. The methyl group of atorvastatin interacts with CYS 291 amino acid residue to form alkyl interactions. In addition, π–π stacking interaction also occurred between HIS 625 amino acid residue and π-electrons of the benzene ring. All these interactions stabilize the atorvastatin-Spike_*Beta*_ complex (Fig. 8B).

We also observed that the atorvastatin binding site is not similar for Spike_*Beta*_ and Spike_*Wild*_complexes. Although atorvastatin was bound at the hind region of Spike_*Beta*_ and Spike_*Wild*_complexes (Figs. 7E and 8A), the binding cavity of the drug has been altered for two proteins (Figs. 7F and 8B). The binding pocket of atorvastatin on Spike_*Beta*_ protein comprised of the residues like GLN 271, PRO 272, ARG 273, THR 274, CYS 291, ALA 292, GLU 298, THR 302, THR 315, SER 316, ASN 317, ARG 319, GLN 321, GLN 628, ASP 627, and HIS 625 (Seq. ID. 271–274, 291–92, 298, 302, 315–21, 625,627, 628). But for Spike*wild-type*, the binding site was composed of the residues, such as ILE312, TYR313, GLN 314, ASN 317, PHE592, LEU849, SER 596, GLY 594, GLN 954, LEU 611, GLN613, ILE670, GLN 965, GLN 957, ALA 958, GLN954, SER1003, THR 1006, TYR1007, THR 961, GLN 1010, and ARG 1014 (Seq. ID. 302, 312–314, 613, 667, 953–961, 849, 1010 and 1014). The residues such as Proline (PRO), arginine (ARG), cystine (CYS), histidine (HIS) were present only in the atorvastatin-Spike_*Beta*_ complex but not in the Spike_*Wild*_-atorvastatin complex.

Interestingly, we also observed that fluvastatin binds at the RBD region of the Spike_*Beta*_ (Fig. 8C,D), with a docking score of −6.05 kcal/mol and MMGBSA score of −15 kcal/mol, respectively. Some significant interactions that stabilize the fluvastatin- pike_*Beta*_-complex include the hydrogen bonds, halogen, and hydrophobic interactions. The carbonyl group (C–O) of the statin forms H-bond with ASN 437, while the hydroxyl groups (OH−) make two H-bonds with SER 375 and TYR 508 residues, respectively. The fluorophenyl group (C_6_H_5_F–) of fluvastatin interacted with ASP 198 and PHE 377 by halogen interactions. Moreover, hydrophobic interactions also occurred due to π-electrons of indole-ring and ALA 372 and ILE 233 residues.

The binding location of fluvastatin was different for Spike_*Beta*_ and Spike_*wild-type*_ proteins. Fluvastatin binds to the hind region of the Spike_*Wild*_ (Fig. 7C,D), but it binds at the RBD (Receptor Binding Domain) region of Spike_*Beta*_protein (Fig. 8C,D). The amino acid residues such as THR 108, THR 114, GLN 115, THR 167, ASP 198, GLY 199, GLY 232, ILE 233, ASN 234, ALA 372, PHE374, SER 375, THR 376, PHE 377, ASN 437, VAL 503, GLY 504, GLN 506, and TYR 508 are present at the binding cavity of Spike_*Beta*_–fluvastatin complex (Fig. 8D). Whereas the residues like THR 302, ILE312, TYR 313, GLN314, THR 315, SER316, LEU 849, GLN 954, GLN957, ALA958, ASN960, THR 961, LYS 964, and GLN613 constitute the binding site of Fluvastatin-Spike_*wild-type*_ complex (Fig. 7D).

##### Spike_*Gamma*_ protein

Docking analysis revealed that pitavastatin has the highest docking score among all statins for the Spike_*Gamma*_ protein. The docking score, glide energy, and MMGBSA scores of pitavastatin against Spike_*Gamma*_ protein are −9.574, −44.458, and −47.18 kcal/mol, respectively (Fig. 8E,F, Table 5). The morphological analysis of the binding site of pitavastatin on Spike_*Gamma*_ reveals the following interactions that stabilize the complex (Fig. 8F). The carboxyl group (C=O–) of the pitavastatin forms an electrostatic salt-bridge with LYS 187, carbonyl group (C–O–) makes two conventional H-bonds with ASN 188 and HIS 207, and the two hydroxyl groups (OH-) forms the same with LEU 176. Additionally, the π-electron cloud of the fluorophenyl group (C_6_H_5_F-) interacts with PHE 175 amino acid residue to form π–π t-shape hydrophobic interactions. Similarly, the π-electrons of the quinoline ring interact with LEU 226 amino acid residue to create π-alkyl interactions (Fig. 8F).

The binding site and location of pitavastatin were also altered for Spike_*Gamma*_and Spike_*wild-type*_ proteins. Pitavastatin was bound to the NTD of the Spike_*Gamma*_protein while it interacted with the hind region of the Spike_*wild-type*_ protein (Fig. 8A,E). The binding site of the pitavastatin-Spike_*Gamma*_complex was comprised of residues like SER 98, ASN99, TRP 104, ASN 121, VAL 126, GLN 173, PRO 174, PHE 175, LEU 176, MET 177, ASP 178, ASN 188, LYS 187, ASN 188, PHE 192, SER 205, LYS 206, and HIS 207 (Fig. 8F). In contrast, the binding site of the pitavastatin-Spike_*wild-type*_ complex consisted of the residues such as THR302, GLN314, TYR313, ILE312, GLY667, ASN 953, GLN954, GLN957, ALA958, ASN 960, THR961, GLN613, LEU849, GLN 1010, and ARG1014 (Fig. 7B).

##### Spike_*Delta*_ protein

Among all the statin molecules, fluvastatin showed the highest docking score against Spike_*Delta*_-protein. The docking score and MMGBSA score of fluvastatin against Spike_*Delta*_ protein were −10.762, −43.705, and −26.84 kcal/mol, respectively (Fig. 8G,H, Table 5). Analysis of the binding site of fluvastatin on Spike_*Delta*_-protein revealed that the carbonyl group (–C–O–) of fluvastatin interacts with ARG 1014 amino acid residue to form salt-bridge interactions. Also, the hydroxyl group (–OH) of fluvastatin interacts with ASN 950, GLN 954, and GLN 1010 amino acid residues to form H-bonds. In addition, the carbon-hydrogen bond (C–H) has also formed between ASN 950 and H-atom of alpha carbon (C–α). The other notable interactions included the alkyl interactions between methyl group (–CH_3_) of fluvastatin and ILE 664 and LYS 310, the π-amide interactions between the π-electrons of indole ring and GLY 946, the π-anion interaction between ASP 663 (polar) and π-electrons of the benzene ring of the drug, and the π-sulfur interactions between the fluorophenyl ring (C_6_H_5_F–) and CYS 662 residue. The π-electron orbital of fluorophenyl ring also formed π-alkyl interaction with LYS 947 and PRO 665 amino acid residues. All these interactions stabilize the Fluvastatin-Spike_*Delta*_-complex (Fig. 8H).

The binding location and cavity of fluvastatin were different for Spike_*Delta*_ protein compared to the Spike_*Wild*_. Fluvastatin was bound at the S2 region of Spike_*Delta*_protein, while it binds at the hind region of Spike_*Wild*_ protein (Figs. 7C, 8G). The binding cavity of the Spike_*Delta*_–fluvastatin complex is comprised of the residues like LYS 310, GLY311, CYS662, ASP 663, ILE 664, PRO 665, SER 943, GLY 946, LYS 947, ASN 950, GLN 954, GLN 1010, and ARG 1014 amino acid residues are present at the binding pocket of (Fig. 8H). Similarly, the amino acids such as THR 302, ILE312, TYR 313, GLN314, THR 315, SER316, LEU 849, GLN 954, GLN957, ALA958, ASN960, THR 961, LYS 964, and GLN613 constituted the binding cavity of Spike_*Wild*_–fluvastatin complex (Fig. 7D).

## Molecular dynamics simulation of fluvastain-target protein complexes

To determine the conformational changes of the target proteins that interact with fluvastatin, we performed MD simulations for 200 ns using Desmond simulation package v6.2 of Schrödinger LLC. The Protein RMSD and ligand RMSD were monitored throughout the simulation period to study the changes in structural conformation of the protein–ligand complex. The Root Mean Square Fluctuation (RMSF) was determined to characterize any local changes along the protein chain due to the binding of fluvastatin. The amino acid residues interacting with the ligand are marked with green-colored vertical bars. Moreover, the interactions between amino acid residues and fluvastatin were monitored throughout the simulation period and represented as 'Simulation Interaction Diagram'. The 'Interactions Fraction' on Y-axis indicates the fraction of simulation time the specific interaction (H-bond, Hydrophobic contact, Ionic interaction, and Water Bridge) was maintained. Values over 1.0 are possible as some protein residue may make multiple contacts of the same subtype with the ligand.

### Helicase–fluvastatin complex

**Figure 9:**
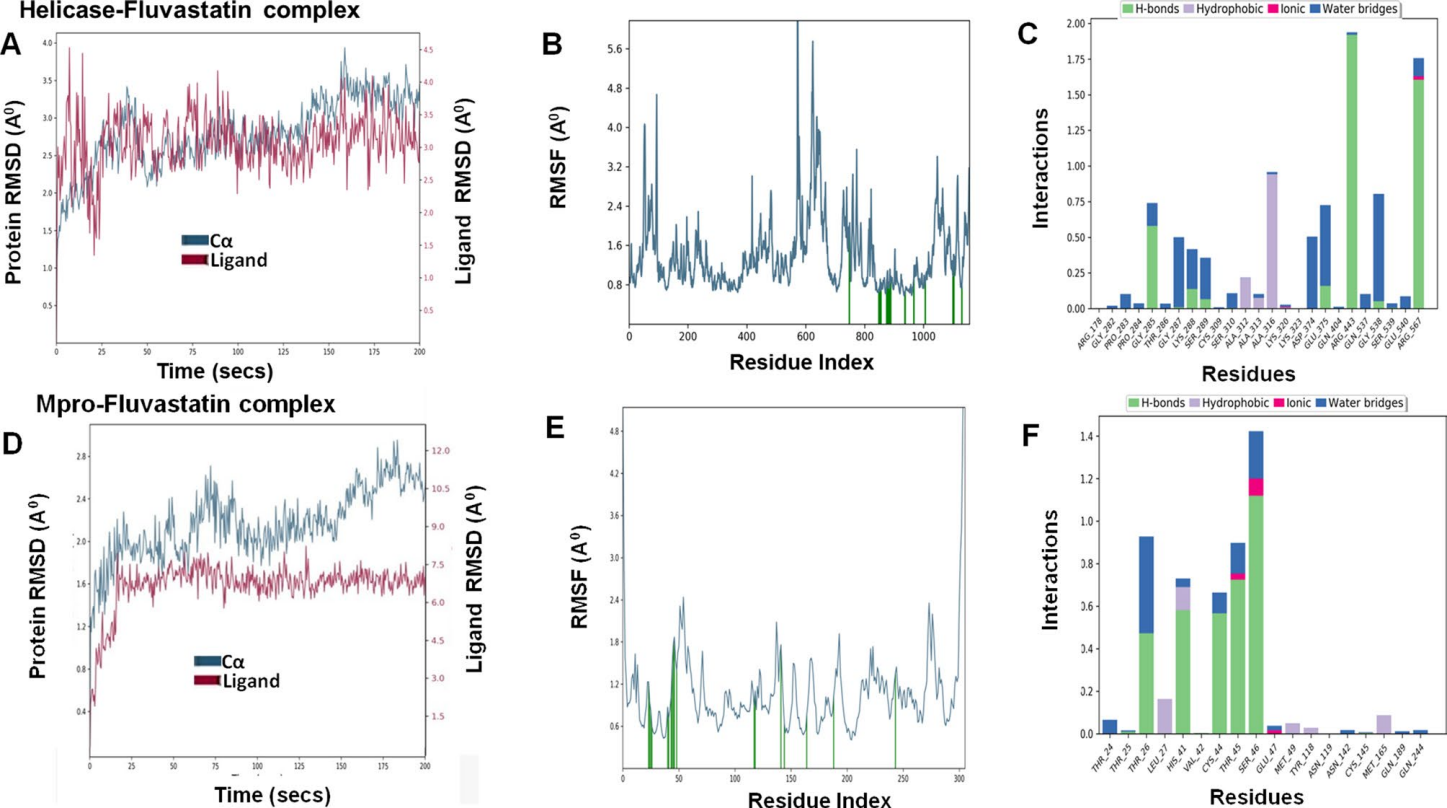
Molecular dynamic simulations of Helicase–fluvastatin complex **(A–C)** and Mpro–fluvastatin complex **(D–F)**, respectively. **(A–C)** RMSD, RMSF, and protein–ligand interactions of helicase–fluvastatin complex, respectively. **(D–F)** RMSD, RMSF, and protein–ligand interactions of Mpro–fluvastatin complex, respectively.

The RMSD value of Helicase in Helicase–fluvastatin complex was 2.79 ± 0.48 Å, whereas RMSD of fluvastatin was 3.07 ± 0.44 (Fig. 9A). The RMSD values got stabilized around the mean values indicating the convergence of simulation. Thus, the complex was stable throughout the study period of 200 ns. As per the RMSF plot, the fluctuations in amino acid residues interacting with fluvastatin (marked with green-colored vertical bars) were the minimum (Fig. 9B). This signifies the stable interaction of fluvastatin with helicase at the binding site throughout the simulation period. Further analysis revealed that the ALA316, ARG443, and ARG567 had the most favorable interactions with fluvastatin throughout the 200 ns period of simulation (Fig. 9C). Interestingly, these residues are the components of the fluvastatin binding site (Fig. 6). The radius of gyration (equivalent to the moment of inertia) of docked fluvastatin was nearly constant (4.29 ± 0.08 Å). All this evidence confirmed the stability of the helicase–fluvastatin complex (Table 6).

**Table 6:** Molecular dynamics analysis of fluvastatin-target protein complex.

Complex name	The radius of gyration (Å) of ligand	SASA (Å)^2^ of ligand	PSA (Å)^2^ of ligand	RMSD	
				Protein C-α (Å)	Ligand with respect to protein (Å)
Mpro–fluvastatin	4.21 ± 0.14	222.82 ± 36.04	165.84 ± 7.84	2.18 ± 0.33	6.95 ± 1.35
RDRP–fluvastatin	4.18 ± 0.16	148.15 ± 30.48	156.3 ± 7.55	1.84 ± 0.25	1.79 ± 0.55
Helicase–fluvastatin	4.29 ± 0.08	130.69 ± 50.70	165.01 ± 7.11	2.79 ± 0.48	3.07 ± 0.44
Spike_Wild_–fluvastatin	4.33 ± 0.14	144.13 ± 40.29	166 ± 7.96	7.2 ± 1.21	6.09 ± 2.02
Spike_Alpha_–fluvastatin	4.43 ± 0.23	274.68 ± 28.69	164.37 ± 8.47	7.05 ± 1.88	5.77 ± 1.56
Spike_Beta_–fluvastatin	4.42 ± 0.12	191.11 ± 38.16	159.35 ± 6.51	4.9 ± 0.72	3.58 ± 1.15
Spike_Gamma_–fluvastatin	4.29 ± 0.14	328.84 ± 63.95	164.3 ± 6.92	4.24 ± 0.71	8.79 ± 2.87
Spike_Delta_–fluvastatin	4.59 ± 0.11	181.25 ± 29.40	160.18 ± 11.61	5.36 ± 1.4	3.13 ± 1.07

### Mpro–fluvastatin complex

The RMSD value of the Mpro protein in the Mpro–fluvastatin complex was 2.18 ± 0.33 Å, whereas the RMSD of fluvastatin was 6.95 ± 1.35 (Fig. 9D). The RMSD values got stabilized around the mean values indicating the convergence of simulation. Thus, the complex was stable throughout the study period of 200 ns. As per the RMSF plot (Fig. 9E), the fluctuations in amino acid residues interacting with fluvastatin (marked with green-colored vertical bars) were the minimum. This signifies the stable interaction of fluvastatin with the protease at the binding site throughout the simulation period. The details of the interactions involved are elaborated in Fig. 9F. The residues such as THR26, SER46, and THR45 had the most favorable interaction with Fluvastatin throughout the simulation period. These amino acids are the components of the fluvastatin binding site (Fig. 4). The radius of gyration (equivalent to the moment of inertia) of docked fluvastatin was nearly constant (4.21 ± 0.14 Å). Thus the results confirmed the stability of the Mpro–fluvastatin complex (Table 6).

### RdRp–fluvastatin complex

**Figure 10:**
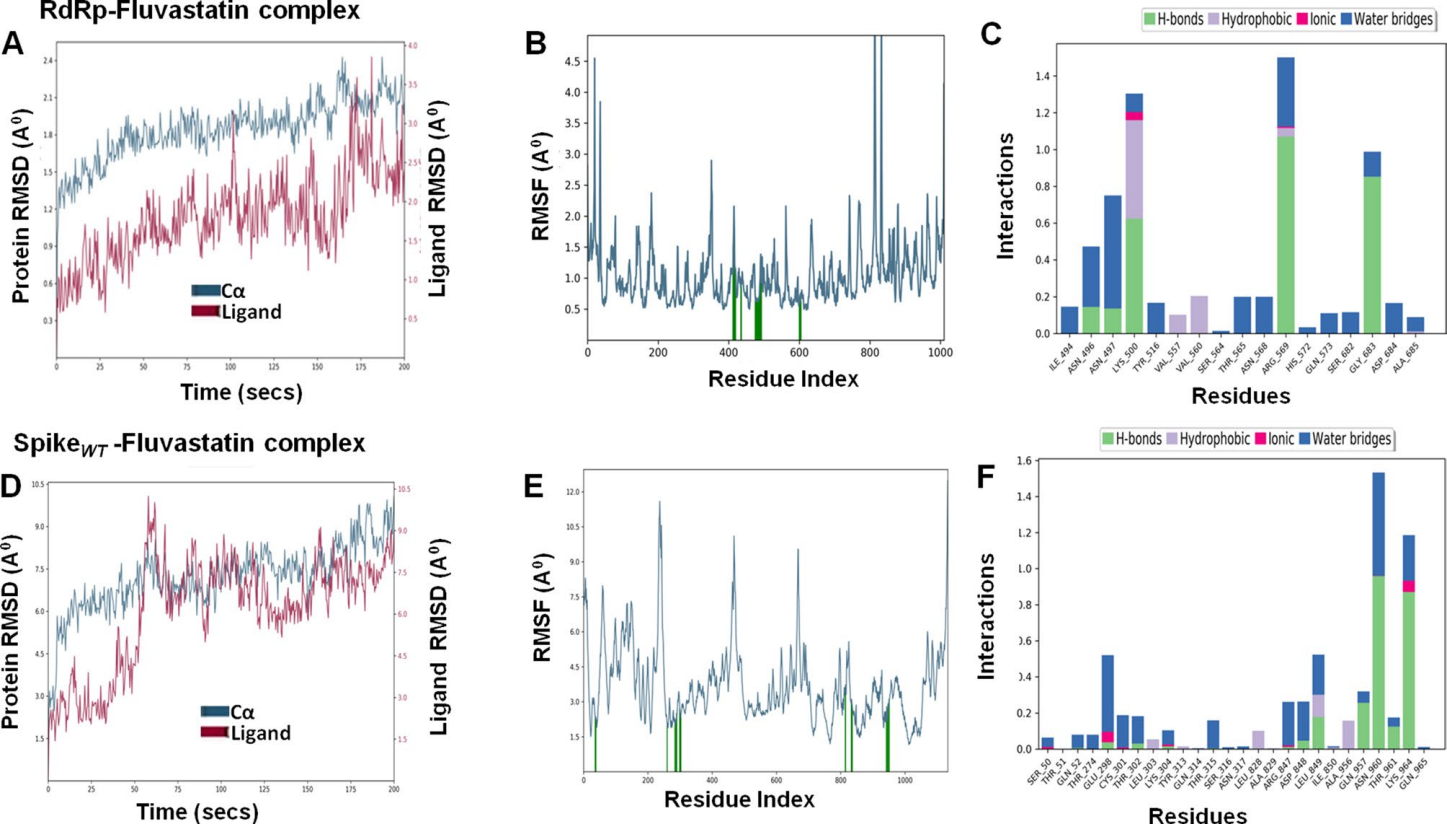
Molecular dynamic simulations of RdRp–fluvastatin complex **(A–C)** and Spike_*WT*_–fluvastatin complex **(D–F)**, respectively. **(A–C)** RMSD, RMSF, and protein–ligand interactions of RdRp–fluvastatin complex, respectively. **(D–F)** RMSD, RMSF, and protein–ligand interactions of Spike_*WT*_–fluvastatin complex, respectively.

The RMSD value of RdRp in the RdRp–fluvastatin complex was 1.84 ± 0.25 Å, whereas RMSD of fluvastatin was 1.79 ± 0.55 (Fig. 10A). The RMSD values got stabilized around the mean values indicating the convergence of simulation. Thus, the complex was stable throughout the study period of 200 ns. As per the RMSF plot (Fig. 10B), the fluctuations in amino acid residues interacting with fluvastatin (marked with green-colored vertical bars) were the minimum, which signifies a stable interaction of fluvastatin with helicase. Also, the residues LYS500, ARG569, and GLY683 showed the most favorable interaction with fluvastatin (Fig. 10C) and constituted the Fluvastatin binding site in RdRp (Fig. 4). The radius of gyration (equivalent to the moment of inertia) of docked fluvastatin was nearly constant (4.18 ± 0.16 Å), which confirmed the stability of the RdRp–fluvastatin complex (Table 6).

### SpikeWT–fluvastatin complex

The RMSD value of SpikeWT in SpikeWT–fluvastatin complex was 7.2 ± 1.21 Å, whereas RMSD of fluvastatin was 6.09 ± 2.02 (Fig. 10D). The RMSD values got stabilized around the mean values indicating the convergence of simulation. The fluctuations in amino acid residues interacting with fluvastatin (marked with green-colored vertical bars) were minimum (Fig. 10E). Also, the residues ASN 960, and LYS964 (present at the binding cavity) are the amino acid residues with which fluvastatin had the most favorable interactions throughout the 200 ns period of simulation (Fig. 10F). The radius of gyration (equivalent to the moment of inertia) of docked fluvastatin was nearly constant (4.33 ± 0.14 Å) (Table 6).

### Spike_*Alpha*_–fluvastatin complex

**Figure 11:**
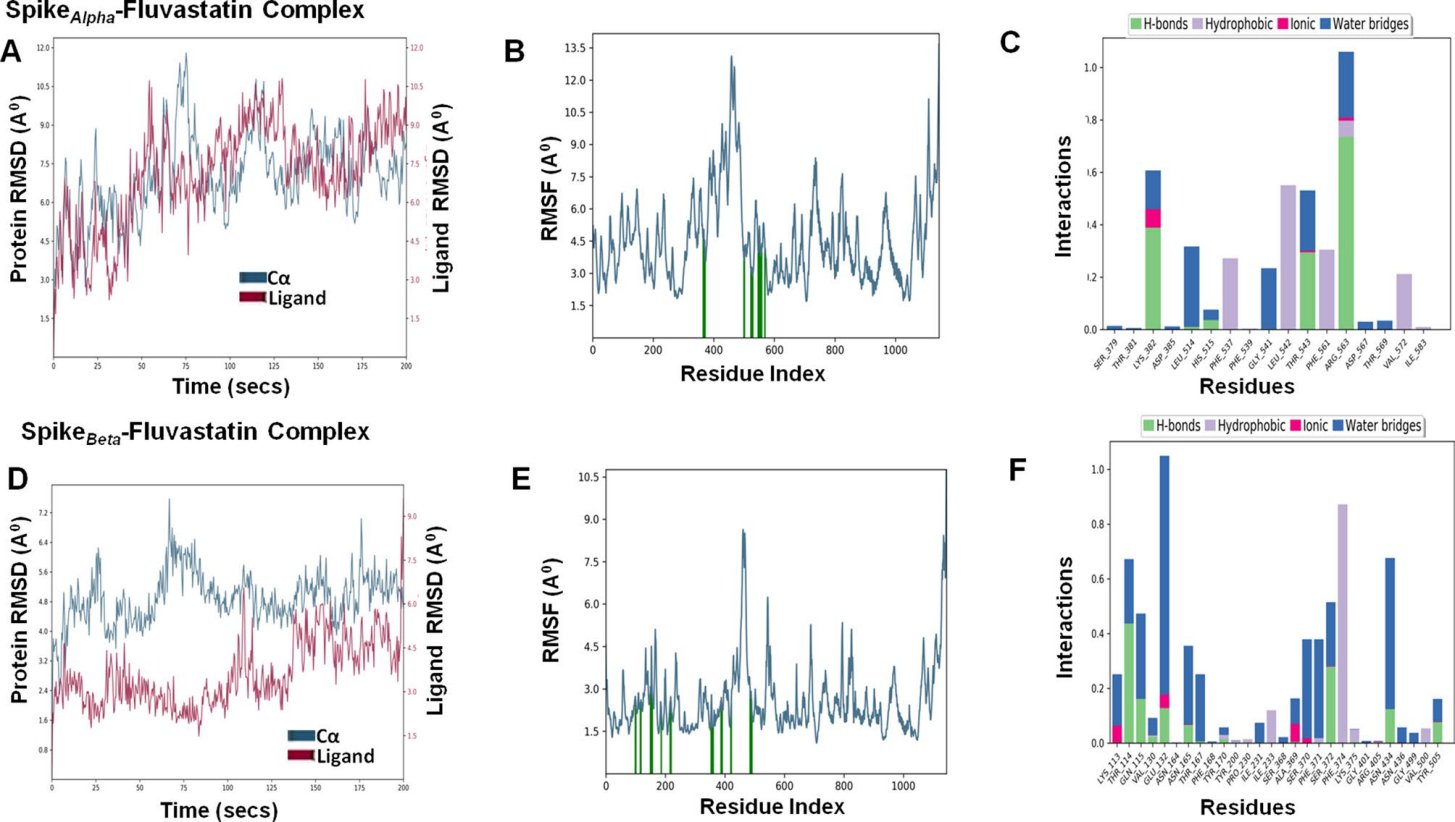
Molecular dynamic simulations of Spike_*Alpha*_–fluvastatin complex **(A–C)** and Spike_*Beta*_–fluvastatin complex **(D–F)**, respectively. **(A–C)** RMSD, RMSF, and protein–ligand interactions of Spike_*Alpha*_–fluvastatin complex, respectively. **(D–F)** RMSD, RMSF, and protein–ligand interactions of Spike_*Beta*_–fluvastatin complex, respectively.

The RMSD value of Spike_*Alpha*_ in Spike_*Alpha*_–fluvastatin complex was 7.05 ± 1.88 Å, whereas RMSD of fluvastatin was 5.77 ± 1.56 (Fig. 11A). The RMSD values got stabilized around the mean values indicating the convergence of simulation and stability of the complex throughout the study period of 200 ns. As per the RMSF plot (Fig. 11B), the fluctuations in amino acid residues interacting with fluvastatin (marked with green-colored vertical bars) were minimal, and also ARG 563 (present at the binding cavity) showed the most favorable interaction with Fluvastatin (Fig. 11C). The radius of gyration (equivalent to the moment of inertia) of docked fluvastatin was nearly constant (4.43 ± 0.23 Å). All the evidence thus confirmed the stability of the Spike_*Alpha*_–fluvastatin complex(Table 6).

### Spike_*Beta*_–fluvastatin complex

The RMSD value of the protein was 4.9 ± 0.72 Å, whereas that of fluvastatin was 3.58 ± 1.15 (Fig. 11D). The RMSD values got stabilized around the mean values indicating the convergence of simulation, indicating the stability of the complex throughout the simulation period. The RMSF for amino acid residues interacting with fluvastatin (marked with green-colored vertical bars) was also minimum (Fig. 11E). This signifies the stable interaction of fluvastatin with Spike_*Beta*_at the binding site throughout the simulation period. Fluvastatin formed water bridges with GLU132 and sustained hydrophobic interactions with PHE374 (Fig. 11F), which constituted the fluvastatin binding site (Fig. 8D). The radius of gyration (equivalent to the moment of inertia) of docked fluvastatin was nearly constant (4.42 ± 0.12 Å). Thus the results confirmed the stability oftheSpike_*Beta*_–fluvastatin complex (Table 6).

### Spike_*Gamma*_–fluvastatin complex

**Figure 12:**
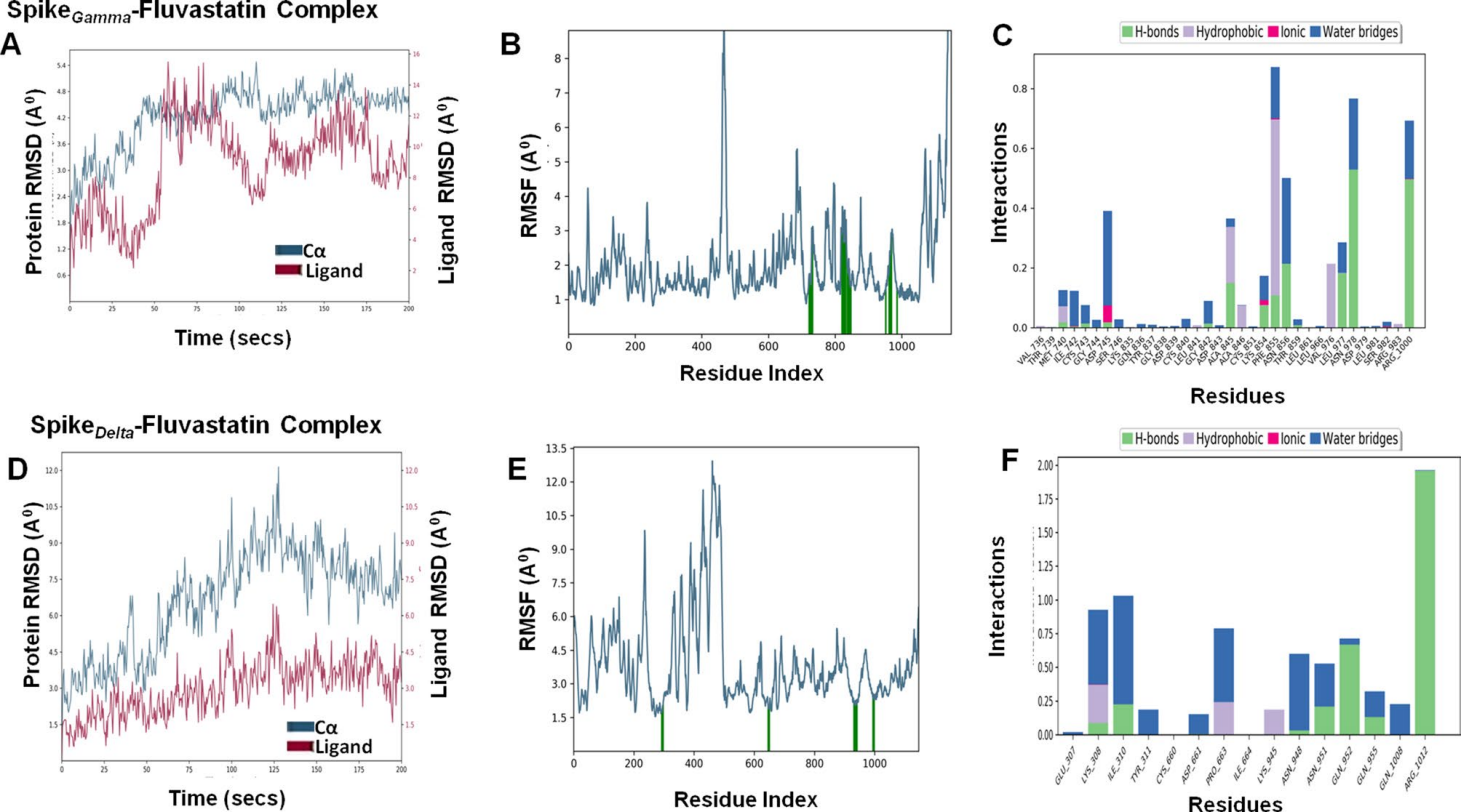
Molecular dynamic simulations of Spike_*Gamma*_–fluvastatin complex **(A–C)** and Spike_*Delta*_–fluvastatin complex **(D–F)**, respectively. **(A–C)** RMSD, RMSF, and protein–ligand interactions of Spike_*Gamma*_–fluvastatin complex, respectively. **(D–F)** RMSD, RMSF, and protein–ligand interactions of Spike_*Delta*_–fluvastatin complex, respectively.

The RMSD value of Spike_Gamma_ was 4.24 ± 0.71 Å, while that of fluvastatin was 8.79 ± 2.87 (Fig. 12A). Although the values rose initially, on the course of the simulation, the RMSD values stabilized and converged, indicating the stability of the complex. As per the RMSF plot, minimal fluctuations were recorded for the amino acid residues interacting with fluvastatin (marked with green-colored vertical bars) (Fig. 12B). Fluvastatin showed the most favorable interactions (H-bond, hydrophobic interactions, and water bridges) with PHE855 and ASN 978 present at the fluvastatin binding site (Fig. 12C). The radius of gyration (equivalent to the moment of inertia) of docked fluvastatin was nearly constant (4.29 ± 0.14 Å). The results thus indicate the stability of the Spike_*Gamma*_–fluvastatin complex (Table 6).

### Spike_*Delta*_–fluvastatin complex

The RMSD value of Spike_*Delt*_a in Spike_*Delta*_ –fluvastatin complex was 5.36 ± 1.4 Å, while that of the ligand was 3.13 ± 1.07 (Fig. 12D). The RMSF plot indicated minimal fluctuations in amino acids that interacted with fluvastatin (marked with green-colored vertical bars) (Fig. 12E). The details of the interactions involved have been elaborated in Fig. 12F and Table 6. The ARG1012 and LYS308 residues shared the most favorable interactions with fluvastatin (Fig. 12F). The radius of gyration (equivalent to the moment of inertia) of docked fluvastatin was nearly constant (4.59 ± 0.11 Å). All this evidence confirmed the stability of the Spike_*Delta*_–fluvastatin complex (Table 6).

## Discussions

Several case–control studies showed a positive association between statin usage and reduced mortality of COVID-19 patients hospitalized with this disease^8,18,89^. Also, it was hypothesized that since statins can up-regulate ACE2 expression^90^, they may prevent coronavirus infection. The direct effect of statins on SARS-CoV-2 was first demonstrated by Gerold et al.^54^. They observed that selected statins, especially fluvastatin, significantly reduced the entry of SARS-CoV-2 into the human respiratory cells and genome copy numbers of SARS-CoV-2 in the infected cells^54^. But the mechanism by which statins can inhibit the SARS-Cov-2 progression is not well understood.

As most of the countries are facing the second or third wave of the pandemic, various vaccines have been commercialized for the people to confer protection against SARS-Cov-2. But, the emergence of VOCs acquiring unique mutations in essential viral genes had raised concern about the effectiveness of the vaccines against these novel variants. In a very recent study, it was reported that the neutralizing antibodies from the individuals, those who received one or two doses of either BNT162b2 or mRNA-1273 vaccines, showed limited efficacy against the pseudoviruses representing the globally predominant VOCs^91^. This observation was supported by other reports revealing the limited effectiveness of the mRNA vaccines or convalescent plasma against the circulating variants^92,93^. Although there are parallel efforts to improve vaccine efficiency against these variants, there is also the necessity of antiviral medications that can target these variants, harboring the concerned mutations.

In our study, nine statin molecules were screened against four selected target proteins of SARS-CoV2 and the mutated S proteins observed in the VOCs, by in silico molecular docking and molecular dynamics study. As per the molecular docking studies, the details of the best-selected candidate drug molecules are tabulated in Tables 4 and 5. The fluvastatin exhibited good binding affinity to all the selected target proteins. It binds with the active site of RdRp (remdesivir binding site), 3CL-Pro (inhibition site), and helicase (ATP binding site). Along with fluvastatin, pitavastatin had shown an equivalent binding affinity for RdRp, and 3-CL-Pro (Table 4). But of all the target proteins, the interaction of fluvastatin with helicase was the best, as a docking score of −11.3 kcal/mol was observed. Analysis of the binding site revealed that fluvastatin binds to the ATP-binding site of helicase. Since helicase plays a pivotal role in the replication of the viral genome, binding of fluvastatin might interfere with the activity of the enzyme resulting in inhibition of the viral replication. This finding might justify the inhibitory effect of fluvastatin on SARS-CoV-2 infection, reported by Gerold et al.^94^. The Fluvastatin-binding site of helicase consisted of a repetitive unit of proline and alanine (PRO 283, PRO 284, ALA 312, ALA 313, and ALA 316), which contributed to ten hydrophobic interactions with the ligand, resulting in the formation of the most stable complex among all the target proteins.

The docking analysis of the statins with the wild-type S-protein and modeled S-mutants demonstrated an interesting trend. Pitavastatin showed maximum binding affinity to Spike wild-type, Spike_*Alpha,*_ and Spike_*Gamma*_ proteins, while Fluvastatin had the maximum affinity for Spike_*Delta*_protein. Interestingly, fluvastatin had fair docking scores for all the S-mutants and showed affinity to bind at the RBD region. Also, the binding location, and the binding cavity of the statins, were altered for the S-mutants, compared to S-wild-type. The analysis of the structural motifs of the S-wild type and the mutants revealed that several posttranslational modifications sites such as the N-glycosylation site and phosphorylation sites emerged or disappeared due to the accumulation of the spike mutations. This may lead to conformational changes in the protein structure, leading to the alteration of binding sites of the statins in the spike-mutants. Alterations in the conformation of the wild-type and mutant spike proteins were also confirmed by the RoseTTAFold server. Further, the MD-simulation studies also indicated that fluvastatin might form stable complexes with the target proteins, including the S-mutants.

**Figure 13:**
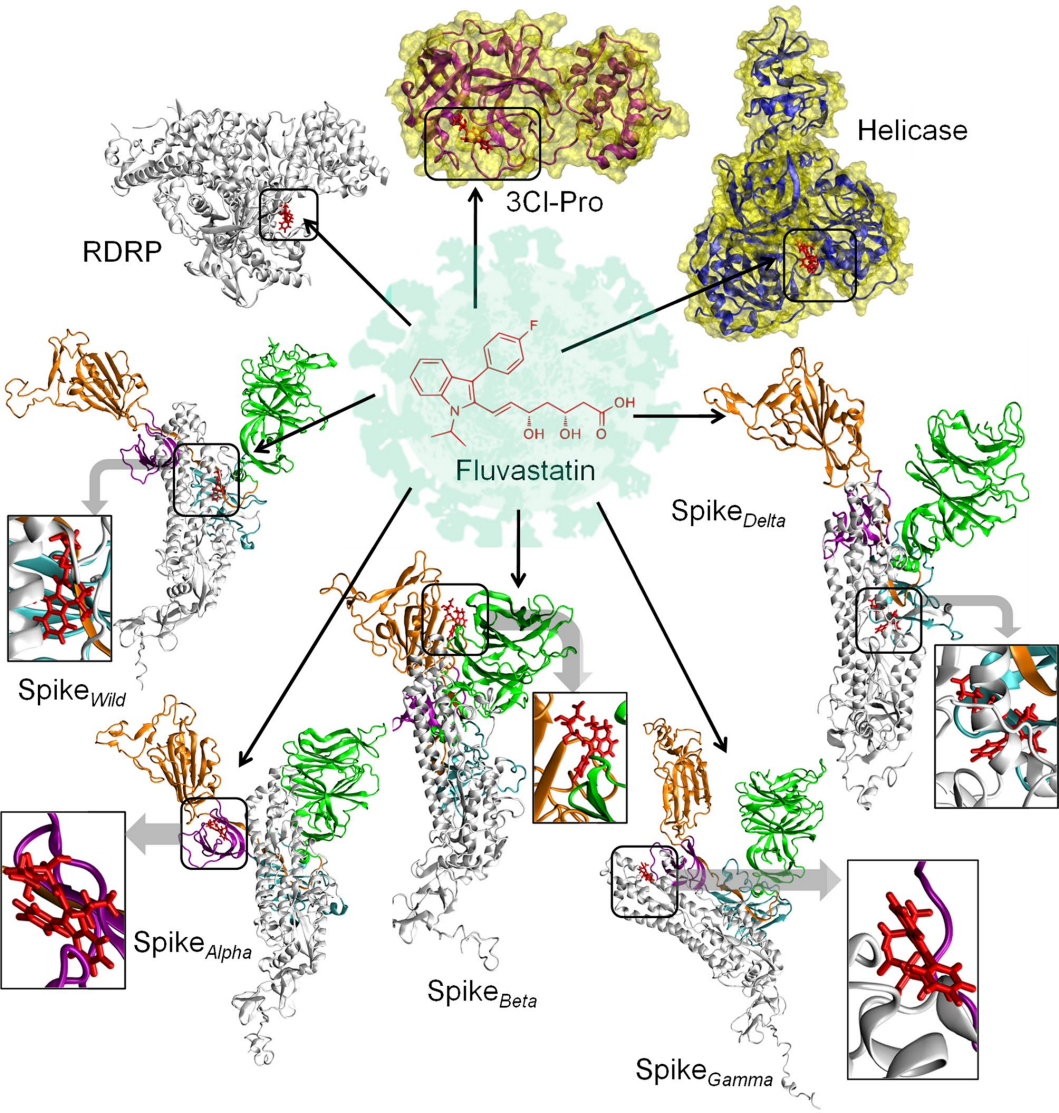
Schematic diagram of the interaction of fluvastatin with the SARS-CoV-2 target proteins. The 2D image of fluvastatin was drawn by ChemSketch, Advanced Chemistry Development, Inc. software (https://www.acdlabs.com/resources/freeware/chemsketch/) and the schematic diagram of COVID-19 virus were drawn by INKSCAPE (version 1.1), an open-source graphics-design software (https://inkscape.org/), used as background. The 3D representation of the protein–ligand complexes were obtained by VMD (visual molecular dynamics) software (version 1.9.3) (https://www.ks.uiuc.edu/Research/vmd/).

Chemical homology, thermodynamic parameters, polarity, and favorable interactions may lead to multiple target sites for a single drug molecule^95,96^. Analysis of the binding sites of fluvastatin in all the target proteins (Table S2), revealed that amino acids like threonine, serine, asparagine, arginine, lysine, and aspartic acid residues were common. Pitavastatin having similar chemical properties like fluvastatin (logP and pKa values) exhibited strong biding affinities to RdRp, 3-CL-Pro, and S-mutants (Fig. 13).

## Conclusion

Although the major limitation of this work is that it is based on a computational prediction without any laboratory validation, such studies are significant for the research groups performing wet-lab experiments intending to identify novel antiviral compounds. The most encouraging result obtained from our study is that fluvastatin emerged as the best drug candidate from the blind docking studies with all the nine statin molecules, which corroborates with the recently published functional study^54,94^. Thus our research will help to predict the molecular mechanism by which this drug inhibits SARS-CoV-2 pathogenesis.

## Supplementary Information

Supplementary Information.
