# Supplementary material for: Computational prediction of the molecular mechanism of statin group of drugs against SARS-CoV-2 pathogenesis
Source: Sci Rep. 2022 Apr 14;12:6241. doi: 10.1038/s41598-022-09845-y (PMC9009757; doi:10.1038/s41598-022-09845-y)
Supplement: Supplementary file 1 — Supplementary Information. [file 41598_2022_9845_MOESM1_ESM.docx]

**Appendix A: Supplementary Materials**


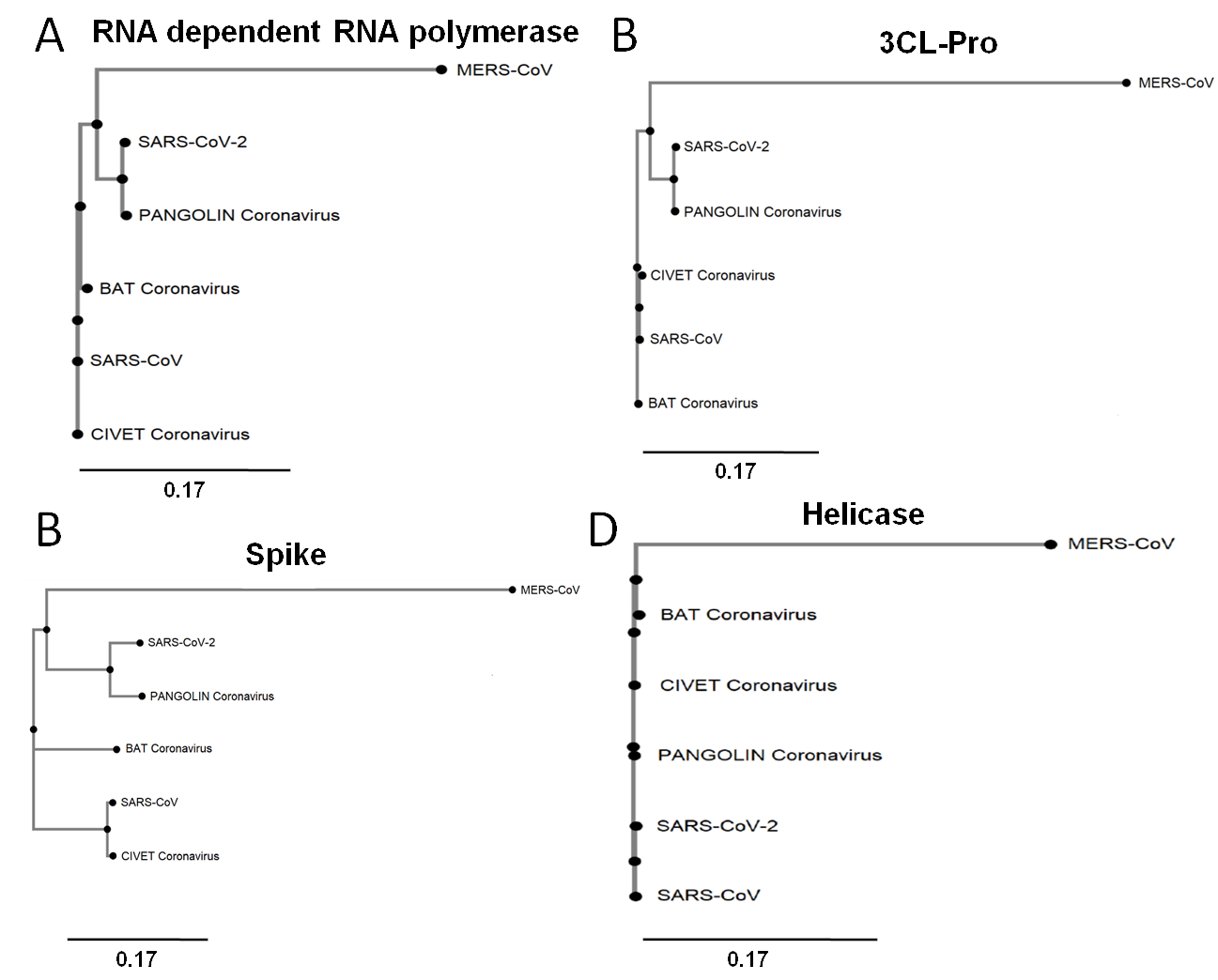


**Figure S1**:Phylogenetic analysis of the SARS-CoV-2 target proteins: A. RNA dependent RNA Polymerase, B: Spike Protein, C. 3-CL-pro, and D: Helicase

**
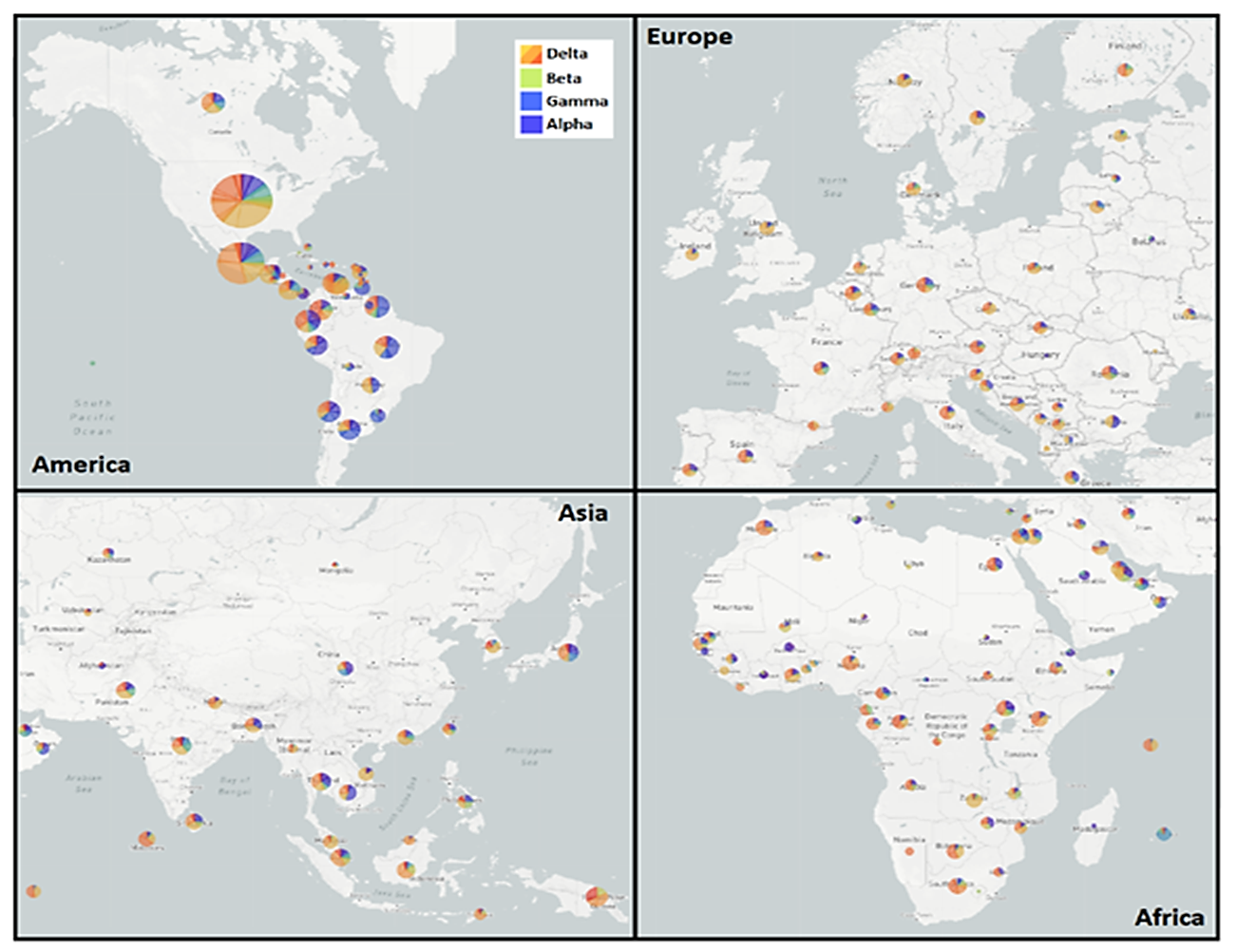
**

**Figure S2 :** Frequencies of major SARS-CoV-2 lineages in different world regions- America, Europe, Asia, and Africa, determined from the SARS-CoV-2 sequences and metadata submitted in GISAID (<https://www.gisaid.org/>), using the Nextstrain ncov pipeline (<https://github.com/nextstrain/ncov>).


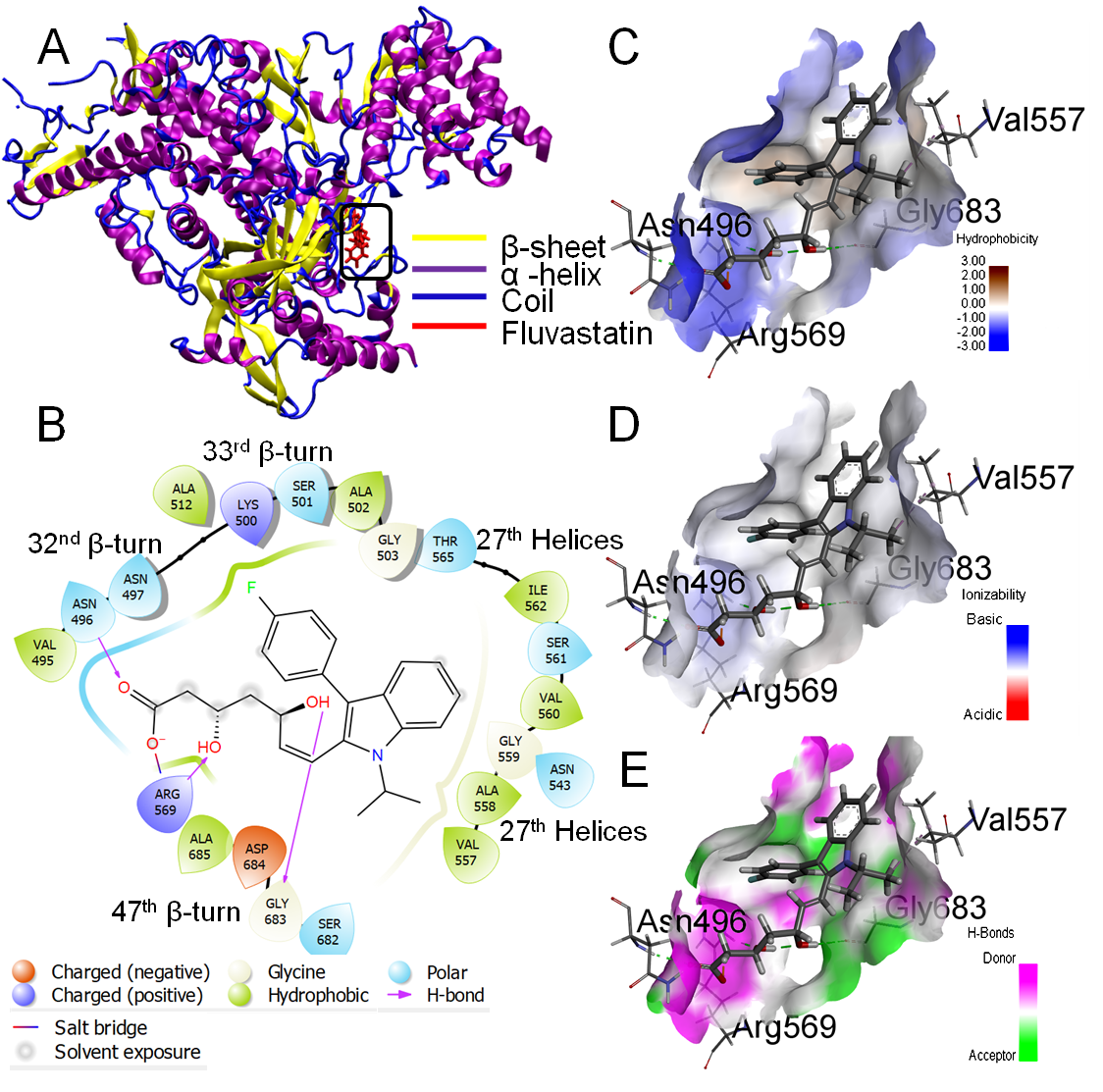


**Figure S3.**Molecular docking study of fluvastatin to RdRp. A: 3D diagram of the ligand-protein complex. B: 2D diagram for representation of interactions of the ligand with the amino acid residues at the binding site. C-E: Mapping of binding site cavity according to hydrophobicity, ionizability, and presence of H-bond donor-acceptor residue, using Discovery Studio Visualizer software, v21.1.0.20298 (<https://discover.3ds.com/discovery-studio-visualizer-download>). All 3D images were generated using the VMD (Visual Molecular Dynamics) software (version 1.9.3) and 2D images were generated by the graphical user interface Maestro, Schrödinger.

(<https://www.schrodinger.com/products/maestro>)

**
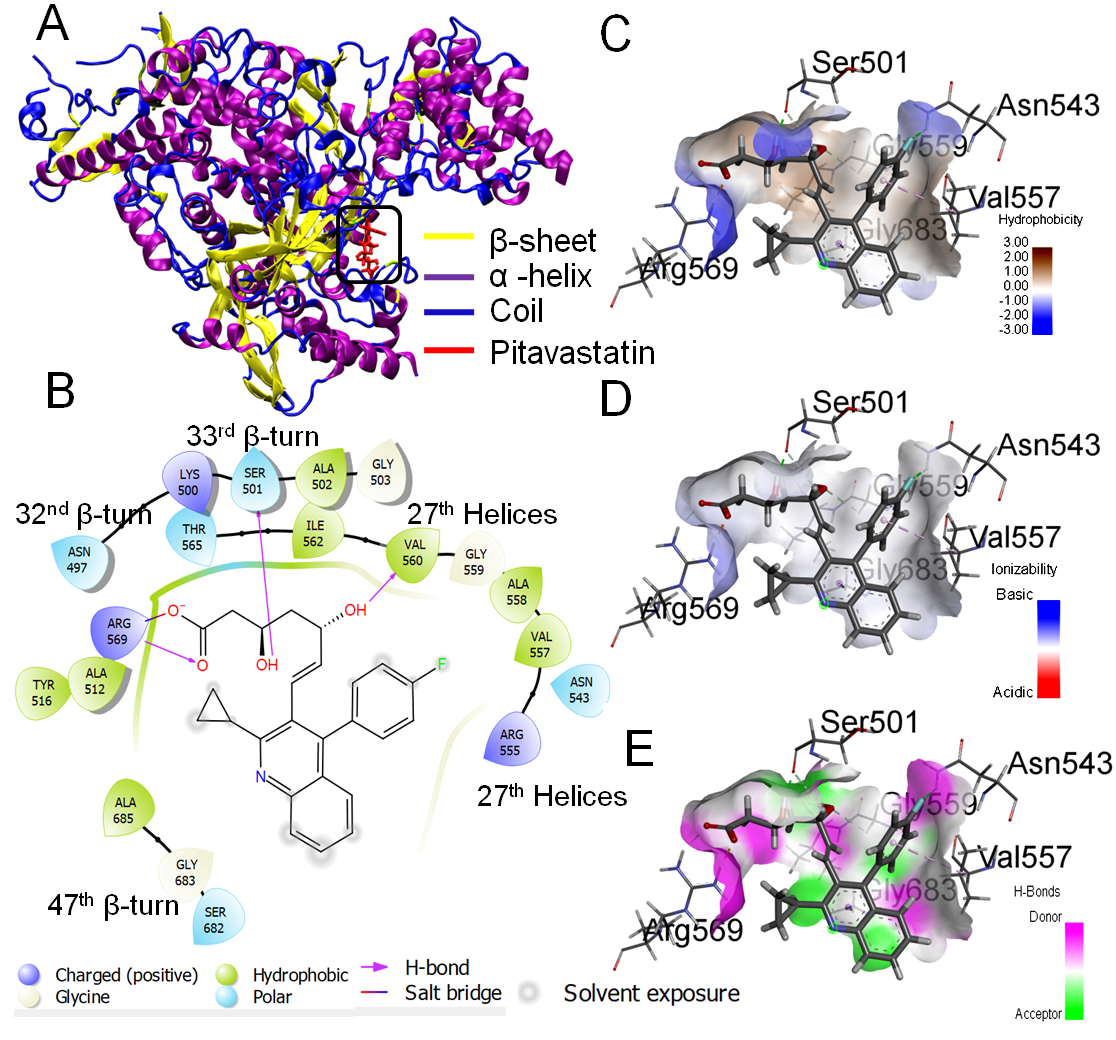
**

**Figure S4**. Molecular docking study of pitavastatin to RdRp. A: 3D diagram of the ligand-protein complex. B: 2D diagram for representation of interactions of the ligand with the amino acid residues at the binding site. C-E: Mapping of binding site cavity according to hydrophobicity, ionizability, and presence of H-bond donor-acceptor residue using Mapping of binding site cavity according to hydrophobicity, ionizability, and presence of H-bond donor-acceptor residue, using Discovery Studio Visualizer software, v21.1.0.20298 (<https://discover.3ds.com/discovery-studio-visualizer-download>). All 3D images were generated using the VMD (Visual Molecular Dynamics) software (version 1.9.3) and 2D images were generated by the graphical user interface Maestro, Schrödinger (<https://www.schrodinger.com/products/maestro>) .


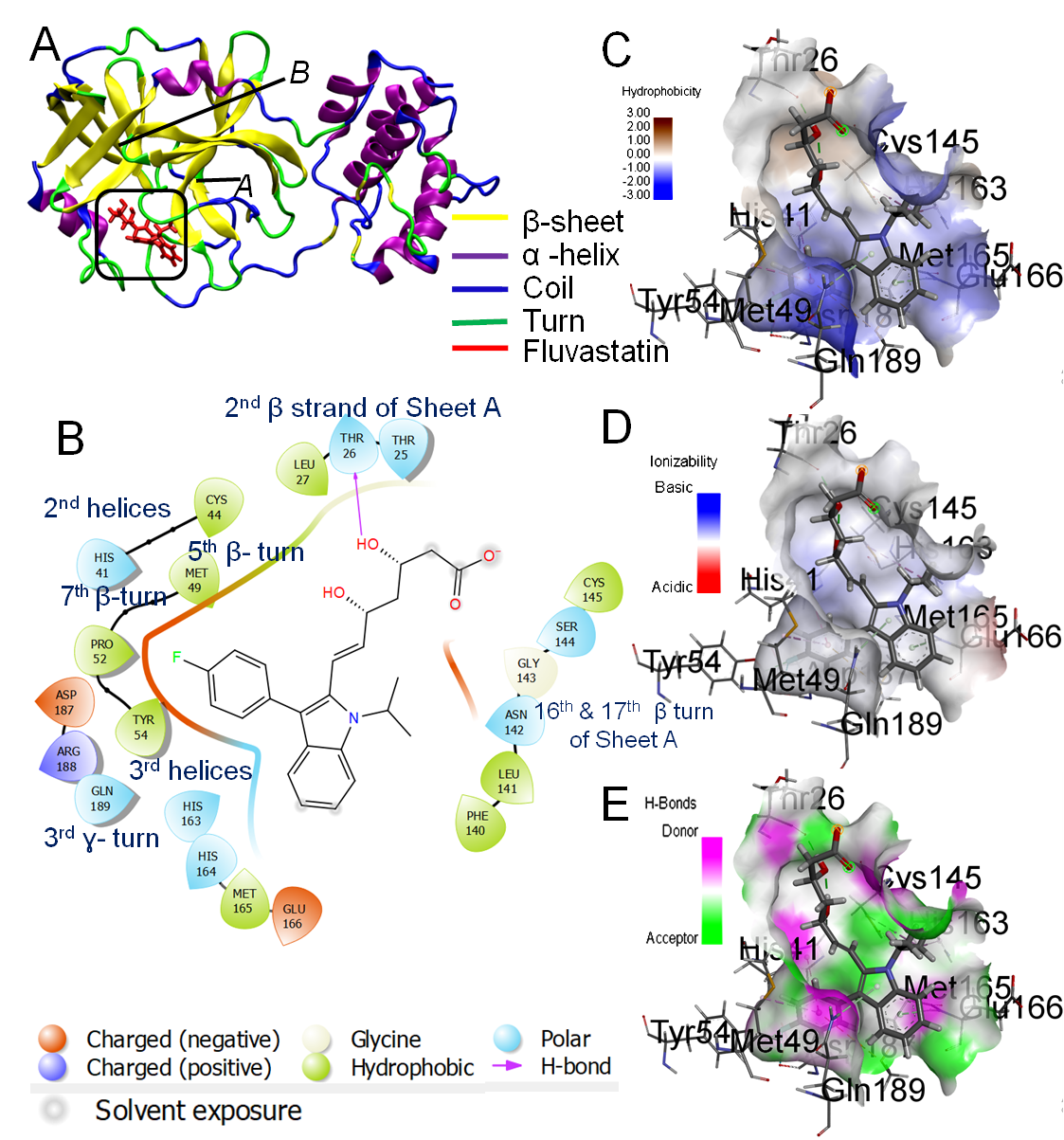


**Figure S5**. Molecular docking study of fluvastatin to 3-Cl-Pro. A: 3D diagram of the ligand-protein complex. B: 2D diagram for representation of interactions of the ligand with the amino acid residues at the binding site. C-E: Mapping of binding site cavity according to hydrophobicity, ionizability, and presence of H-bond donor-acceptor residue, using Discovery Studio Visualizer software, v21.1.0.20298 (<https://discover.3ds.com/discovery-studio-visualizer-download>). All 3D images were generated using the VMD (Visual Molecular Dynamics) software (version 1.9.3) and 2D images were generated by the graphical user interface Maestro, Schrödinger.

(<https://www.schrodinger.com/products/maestro>).


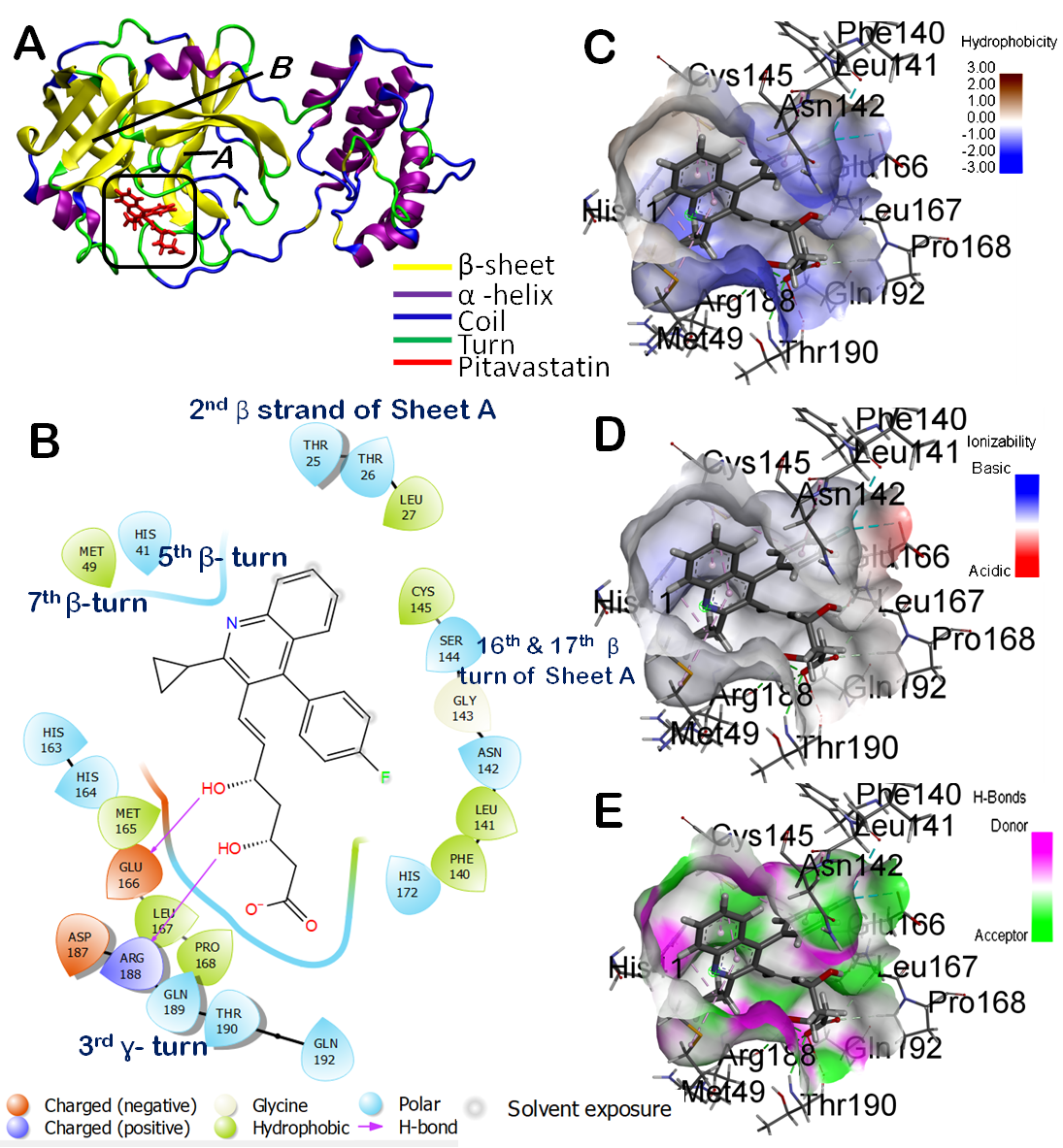


**Figure S6**. Molecular docking study of pitavastatin to 3-Cl-Pro. A: 3D diagram of the ligand-protein complex. B: 2D diagram for representation of interactions of the ligand with the amino acid residues at the binding site. C-E: Mapping of binding site cavity according to hydrophobicity, ionizability, and presence of H-bond donor-acceptor residue,using Discovery Studio Visualizer software, v21.1.0.20298 (<https://discover.3ds.com/discovery-studio-visualizer-download>). All 3D images were generated using the VMD (Visual Molecular Dynamics) software (version 1.9.3) and 2D images were generated by the graphical user interface Maestro, Schrödinger (<https://www.schrodinger.com/products/maestro>) .


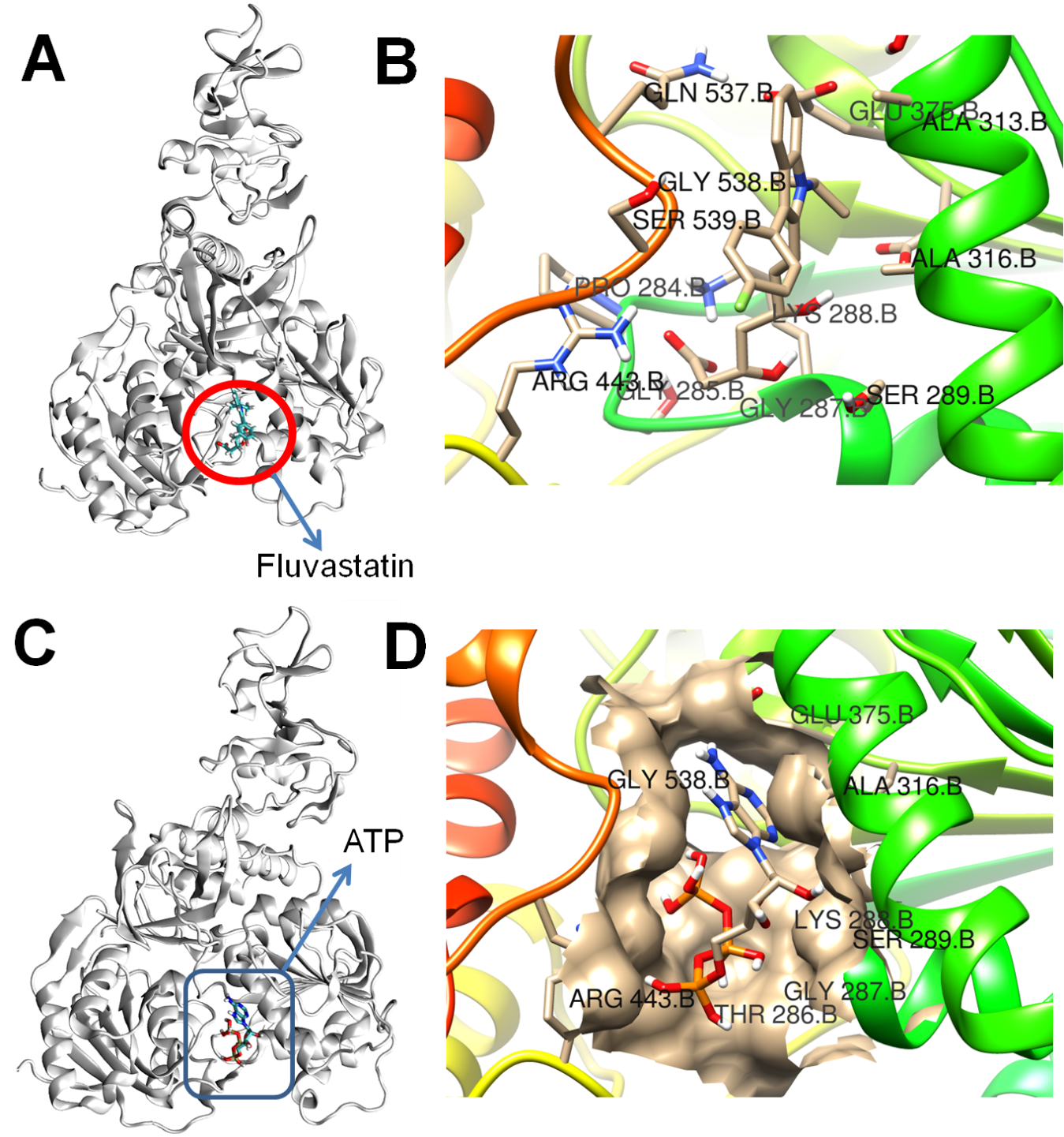


**Figure S7**. The binding site of fluvastatin (**A and C**) and ATP (**B and D**) on helicase, determined by Chimera Software (<https://www.cgl.ucsf.edu/chimera/>)

**Table S1: Physicochemical properties of all statin molecules.**

| **Statins** | **Pub Chem CID** | **Molecular weight** | **Solubility**  **(**μg/mL) | **TPSA (Topological Polar Surface Area) (Å^2^)** | **Hydrogen Bond Donor Count** | **Hydrogen Bond Acceptor Count** | **logP** | **pKa** |
| --- | --- | --- | --- | --- | --- | --- | --- | --- |
| Atorvastatin | 60823 | 558.6 | 1.12 | 111.79 | 4 | 6 | 6.36 | 4.46 |
| Cerivastatin | 446156 | 459.5 | 240 | 99.88 | 3 | 7 | 3.4 | pKa1 = 3.9  pKa2 = 5.4 |
| Fluvastatin | 446155 | 411.5 | 460 | 82.69 | 3 | 5 | 4.85 | 4.5 |
| Lovastatin | 53232 | 404.5 | 0.4 | 72.83 | 1 | 5 | 4.68 | 13.49 |
| Mevastatin | 64715 | 390.5 | 1.4 | 72.83 | 1 | 5 | 3.95 | 14.91 |
| Pitavastatin | 5282452 | 421.5 | 0.426 | 90.65 | 3 | 6 | 4.8 | 4.3 |
| Pravastatin | 54687 | 424.5 | 6 | 124.29 | 4 | 7 | 2.18 | 4.2 |
| Simvastatin | 54454 | 418.6 | 0.03 | 72.83 | 1 | 5 | 4.68 | 14.91 |

**Table S2: Similarity of amino acids comprising the binding sites of fluvastatin at different target proteins.**

| **Target Protein** | **Amino acids at binding site** | **Interaction types** | **Bond distance (Å)** | **Part of fluvastatin molecule involved in the interaction** |
| --- | --- | --- | --- | --- |
| 3CL-Pro | ASN142 | H-bond Donor | 1.69432 | O4 |
|  |  |  | 2.80973 | O4 |
|  |  |  | 2.58498 | O1 |
|  | SER144 |  | 2.37891 | O3 |
|  | THR25 |  | 3.0612 | O2 |
|  | ASP187 | Halogen acceptor | 3.63138 | F1 |
| Helicase | LYS283 | H-Donor | 1.81859 | O4 |
|  |  |  | 1.78696 | O4 |
|  |  |  | 2.33093 | O3 |
|  | SER284 |  | 2.71129 | O1 |
|  |  |  | 1.66841 | O2 |
|  | PRO279 |  | 2.8378 | O3 |
|  | SER284 |  | 2.35577 | O1 |
|  |  |  | 2.8405 | O2 |
| RdRp | ARG489 | Electrostatic (Attractive Charge) | 2.9811 | O4 |
|  | ASN417 | Conventional Hydrogen Bond | 1.92779 | O4 |
|  | ARG489 |  | 1.82987 | O3 |
|  | ARG489 |  | 2.85354 | O2 |
|  | ASP604 | Carbon Hydrogen Bond | 2.64315 | H10 |
|  | THR485 | π -σ | 2.81061 | π-Orbitals |
|  | LYS420 | Alkyl | 4.28062 | C3 |
|  |  | π -Alkyl | 4.43519 | π -Orbitals |
|  |  | π-interaction | 5.2991 | π -Orbitals |
|  |  | Salt Bridge; Attractive Charge | 2.09659 | O4 |
|  |  | Conventional Hydrogen Bond | 1.89781 | O2 |
| Spike*_Wild_* | LYS 964 | Salt-bridge | 1.77 | O3 |
|  | ASN 960 | Conventional H-bond | 1.68141 | H11 |
|  | ASN 960 | Conventional H-bond | 1.9915 | H15 |
| Spike*_Alpha_* | ARG 567 | Salt-bridge | 2.1345 | O4 |
|  |  | Electrostatic interaction | 3.36119 | O3 |
|  |  | Conventional H bond | 2.611 | O2 |
| Spike*_Beta_* | ASN 437 | Conventional H bond | 2.4276 | O4 |
|  | ASP 198 | Halogen interaction | 3.0424 | F1 |
| Spike*_Gamma_* | ARG 1000 | Salt-bridge | 2.07 | O4 |
|  |  | Salt-bridge | 1.9435 | O3 |
|  | ASN 856 | Conventional H bond | 2.0278 | H11 |
|  | ASP 745 | π-anionic interaction | 3.44 | π-Orbitals |
| Spike*_Delta_* | ARG 1014 | Salt-bridge | 1.89 | O3 |
|  |  | Conventional H bond | 2.395 | O4 |
|  | ASN 950 | Conventional H bond | 1.847 | O2 |
|  | LYS 310 | Alkyl | 3.846 | Alkyl group (C1) |
|  |  |  | 4.38 | Alkyl group (C3) |
|  | LYS 947 | π- Alkyl | 5.25 | π-Orbitals |
